# A meta-analytic reliability generalization study of the Bedtime Procrastination Scale

**DOI:** 10.3389/fpsyg.2026.1709258

**Published:** 2026-02-18

**Authors:** Esra Oyar, Serpil Çelikten-Demirel, Ayşenur Erdemir

**Affiliations:** 1 Department of Measurement and Evaluation in Education, Gazi University, Ankara, Türkiye; 2 Department of Measurement and Evaluation in Education, Dicle University, Diyarbakır, Türkiye; 3 Turkish National Police Academy, Ankara, Türkiye

**Keywords:** Bedtime Procrastination Scale, Cronbach’s alpha, McDonald’s omega, meta-analysis, reliability generalization

## Abstract

**Introduction:**

Bedtime procrastination is defined as deliberately delaying sleep without any external conditions preventing sleep. One of the most frequently used scales in this field is the Bedtime Procrastination Scale (BPS). The original form of the scale consists of nine items rated on a 5-point Likert scale. The BPS is a measurement tool that has been applied to many cultures, both in the language in which it was developed and in adaptations to different languages. This study aims to examine the reliability coefficients obtained from different studies for the BPS using meta-analysis methods and to determine the average effect size for the scale.

**Method:**

For this purpose, studies were searched in the Scopus, Proquest, Web of Science, ScienceDirect, EBSCO, and Google Scholar databases between 2014 and 2025 using the keyword “Bedtime Procrastination Scale,” and analyses were performed on 128 reliability coefficients (127 for *α* and 11 studies for *ω*). The Bonnet transformation was used to obtain the average reliability coefficient.

**Results:**

Cronbach’s alpha (*α*) was estimated at 0.855 [95% CI (0.843, 0.865)], and McDonald’s omega (*ω*) was estimated at 0.867 [95% CI (0.834, 0.894)]. There was no publication and reporting bias found for either reliability coefficient analysis; however, the magnitude of heterogeneity suggests that moderator analyses are warranted to explain systematic variability across studies. The moderator analysis found that the variables mean age, SD age, region, and sample group were significant for the Cronbach alpha coefficient, while only the sample group variable was significant for the McDonald’s omega coefficient.

**Discussion:**

Overall, the findings indicate that the Bedtime Procrastination Scale demonstrates high and acceptable reliability across studies for both Cronbach’s alpha and McDonald’s omega. While age, region, and sample type emerged as significant moderators (for Cronbach’s alpha), a substantial proportion of heterogeneity remained unexplained, indicating that reliability variability cannot be attributed to a single set of study characteristics. Although reliability was generally adequate, the observed heterogeneity and wide prediction intervals suggest that caution is warranted when the scale is used in high-stakes or critical decision-making contexts. Moreover, recommendations were made for both researchers and practitioners.

## Introduction

Procrastination is a common phenomenon that occurs frequently in daily life and is extensively studied. In the scholarly literature, procrastination is characterized as the voluntary postponement of intended tasks or decisions, even when individuals anticipate that such delays will be detrimental to desired outcomes (Steel, 2007; Sirois and Pychyl, 2013).

Procrastination occurs across multiple life domains, including academic contexts (Schouwenburg, 1995; Klingsieck, 2013; Steel, 2007), occupational settings (Nguyen et al., 2013), everyday household tasks (Ferrari et al., 1995; Milgram et al., 1988), and health-related behaviors (Sirois, 2004; Sirois, 2007), where individuals delay necessary tasks in ways that may undermine performance and wellbeing.

Beyond these domains, procrastination has also been increasingly examined in relation to sleep-related behaviors. Research over the past decade has consistently shown that insufficient or poor-quality sleep is associated with a range of adverse mental and physical health outcomes. Bedtime procrastination, as a self-regulatory failure that prioritizes leisure or technology-related activities over sleep, has been identified as a key behavioral contributor to sleep deprivation, leading to reduced sleep duration and impaired sleep quality (Chung et al., 2020; Kroese et al., 2014; Kühnel et al., 2016). In contemporary societies characterized by fast-paced lifestyles (Åkerstedt and Nilsson, 2003; Basner et al., 2007) and pervasive use of digital technology (Cain and Gradisar, 2010; Exelmans and Bulck, 2017), bedtime procrastination has emerged as a prevalent and consequential behavior with significant implications for mental and physical health (Ford and Kamerow, 1989; Howarth and Miller, 2024; Manocchia et al., 2001; Zhang et al., 2024). In light of the growing recognition of bedtime procrastination as a significant determinant of sleep-related outcomes, it is imperative to employ a reliable and valid instrument to measure this construct. Furthermore, the existing measurement tool must be subjected to rigorous evaluation in terms of its validity and reliability.

### Bedtime procrastination scale

Bedtime procrastination was defined as the voluntary delay of going to bed without external circumstances preventing sleep. The Bedtime Procrastination Scale (BPS), originally developed in English (Kroese et al., 2014), is a widely used instrument to assess this construct. The instrument comprises nine items within a unidimensional structure, four of which are reverse-worded. Over the past decade, the BPS has been translated and adapted into several languages, including Arabic (Hammoudi et al., 2021), Chinese (Ma et al., 2021), German (Bernecker and Job, 2019), Spanish (Brando-Garrido et al., 2022), Dutch (Broers, 2014), Korean (An et al., 2019), Japanese (Miyagawa et al., 2024), Polish (Herzog-Krzywoszanska and Krzywoszanski, 2019), Portuguese (Magalhães et al., 2020), Indonesian (Rahayu and Caninsti, 2024), Persian (Rasouli et al., 2025), and Turkish (Dinç et al., 2016). With these various versions, the BPS has been administered across a wide range of cultural contexts, including Western (e.g., the USA and Germany), East Asian (e.g., China and Japan), South Asian (e.g., India and Pakistan), Middle Eastern (e.g., Iran and Saudi Arabia), and Southeast Asian societies (e.g., Singapore and Indonesia). In addition, studies employing the BPS have targeted populations with age ranges spanning from early adolescence to adulthood, with approximately 13–56 years (Exelmans and Bulck, 2021; Kullik et al., 2025). These populations include young adults (Flores et al., 2023; Jeoung et al., 2023), adolescents (Deng et al., 2024; Pu et al., 2022), university students (Hamvai et al., 2023; Xu et al., 2024), and the general population (Deng et al., 2022; Miyagawa et al., 2024).

This extensive use underscores the widespread acceptance and versatility of BPS as a measure of bedtime procrastination. However, the extensive use of the BPS across diverse populations and research contexts also raises important questions regarding the consistency and generalizability of its reliability estimates. Despite its widespread use, the reliability of its scores has shown substantial inconsistency across studies, with reported internal consistency estimates (e.g., Cronbach’s alpha and McDonald’s omega) ranging from 0.540 to 0.982. Moreover, several studies have relied on reliability coefficients reported in the original validation study rather than estimating reliability from their own data, a practice referred to as reliability induction (Thompson and Vacha-Haase, 2000; Huang et al., 2023; Manasa and Saju Stephen, 2024; Son and Kwag, 2021). This practice reflects the erroneous assumption that reliability is an invariant property of the instrument itself, rather than a characteristic of the scores obtained within a specific sample and research context (Thompson and Vacha-Haase, 2000). Given the diversity of samples and study conditions under which the BPS has been administered, variability in reported reliability estimates is expected, and the absence of consistently reported coefficients further limits conclusions regarding the scale’s psychometric robustness.

The widespread use of the Bedtime Procrastination Scale across multiple languages and diverse target populations, together with the observed variability and lack of stability in its reported reliability coefficients across studies, was explicitly highlighted as the research gap that justifies conducting a reliability generalization meta-analysis (RGMA) of the BPS.

### Meta-analytical reliability generalization

Reliability is a fundamental psychometric property of test scores, reflecting the consistency of scores across administrations under comparable conditions, yet it may vary across applications as a function of score variability, sample characteristics, and administration procedures (Crocker and Algina, 1986; Henson and Thompson, 2002). Considering that reliability coefficients can fluctuate across different administrations, systematically identifying the factors that influence—or do not influence—this variability within the context of a given measurement instrument can inform the implementation of more rigorous and precise reliability practices in subsequent research employing the instrument (Henson and Thompson, 2002; López-Ibáñez et al., 2024; Vacha-Haase et al., 2002). In line with American Psychological Association’s (2020) Journal Article Reporting Standards, researchers are explicitly encouraged to estimate and report reliability coefficients for the scores analyzed in their own samples, underscoring that reliability is a property of the obtained scores rather than a fixed characteristic of the measurement instrument (Appelbaum et al., 2018). Fundamentally developed for this purpose, reliability generalization (RG) studies aim to (a) examine the distribution of reliability coefficients reported in the literature, (b) identify possible sources that account for variability in these estimates, and (c) provide pooled reliability estimates for the instrument under investigation (López-Ibáñez et al., 2024; Vacha-Haase, 1998; Vacha-Haase et al., 2002). Through the use of RG, researchers can potentially design future studies in ways that enhance score reliability, increase effect sizes, improve statistical power, and strengthen the likelihood of obtaining significant results (Henson and Thompson, 2002).

To summarize, the widespread use of the Bedtime Procrastination Scale, along with the non-constant nature of its reliability and the variability of reliability coefficients reported across studies, underscores the importance of conducting a reliability generalization meta-analysis for this instrument. Such an approach enables a systematic evaluation of the psychometric robustness of the BPS, ensuring its valid and reliable application across diverse populations and research contexts.

### Purpose of the study

The purpose of the present study is to examine the meta-analytic reliability of the BPS while considering various moderator variables, thereby accounting for the heterogeneity observed in reliability coefficients.

By applying this approach to the BPS, the present study aims to (a) quantify the overall reliability of the scale across published research, (b) evaluate the variability in reliability estimates, and (c) explore predictors of heterogeneity of the reliability, with continuous moderators including the mean age, standard deviation of age, sample size, percentage of female, mean of BPS scale score and standard deviation of BPS scale score by the way categorical moderators including region, language, sample type and publication type.

## Method

A Reliability Generalization Meta-Analysis (RGMA; Vacha-Haase, 1998) was conducted to estimate average population reliability coefficients for the BPS. The conduct and reporting followed the REGEMA guidelines and checklist proposed by Sánchez-Meca et al. (2021), developed to address the lack of dedicated RG standards. These guidelines aim to enhance clarity, reproducibility, and transparency in RG studies through a structured flow and a 30-item checklist across eight dimensions, which informed study selection, coding, and synthesis.

### Search strategy

In the first stage of the data collection process, a literature search was conducted in the Scopus, Proquest, Web of Science, ScienceDirect, EBSCO, and Google Scholar databases using the keyword “Bedtime Procrastination Scale.” These searches were conducted in July 2025, and no year limit was set for the studies. Since the scale addressed in the study was developed in 2014, the studies reviewed cover the period between 2014 and July 2025. Hand-searching was also performed.

### Inclusion and exclusion criteria

In the process of including studies in the meta-analysis, (1) the study must have been published as an article or thesis; (2) the language of publication must be English; (3) the scale used in the study must be the Bedtime Procrastination Scale (BPS) developed by Kroese et al. (2014) or adapted versions; and (4) the reliability coefficient and sample size related to the BPS must be reported in the study.

On the other hand, studies were excluded (1) if the studies were conducted using a qualitative research design, bibliometric studies, meta-analyses, or systematic reviews; (2) if the items on the BPS scale were reduced or additional items were added; (3) if the BPS scale was used with a rating scale other than a 5-point Likert scale; (4) if the study was written in a language other than English, (5) if the study was published in a format other than an article or thesis, (6) if the study used data from a previously included study (same sample), and (7)sStudies that used the BPS but either explicitly reported no reliability coefficients (by report) or omitted them altogether (by omission), and did not respond to requests for reliability information or an anonymized data set.

**Figure 1:**
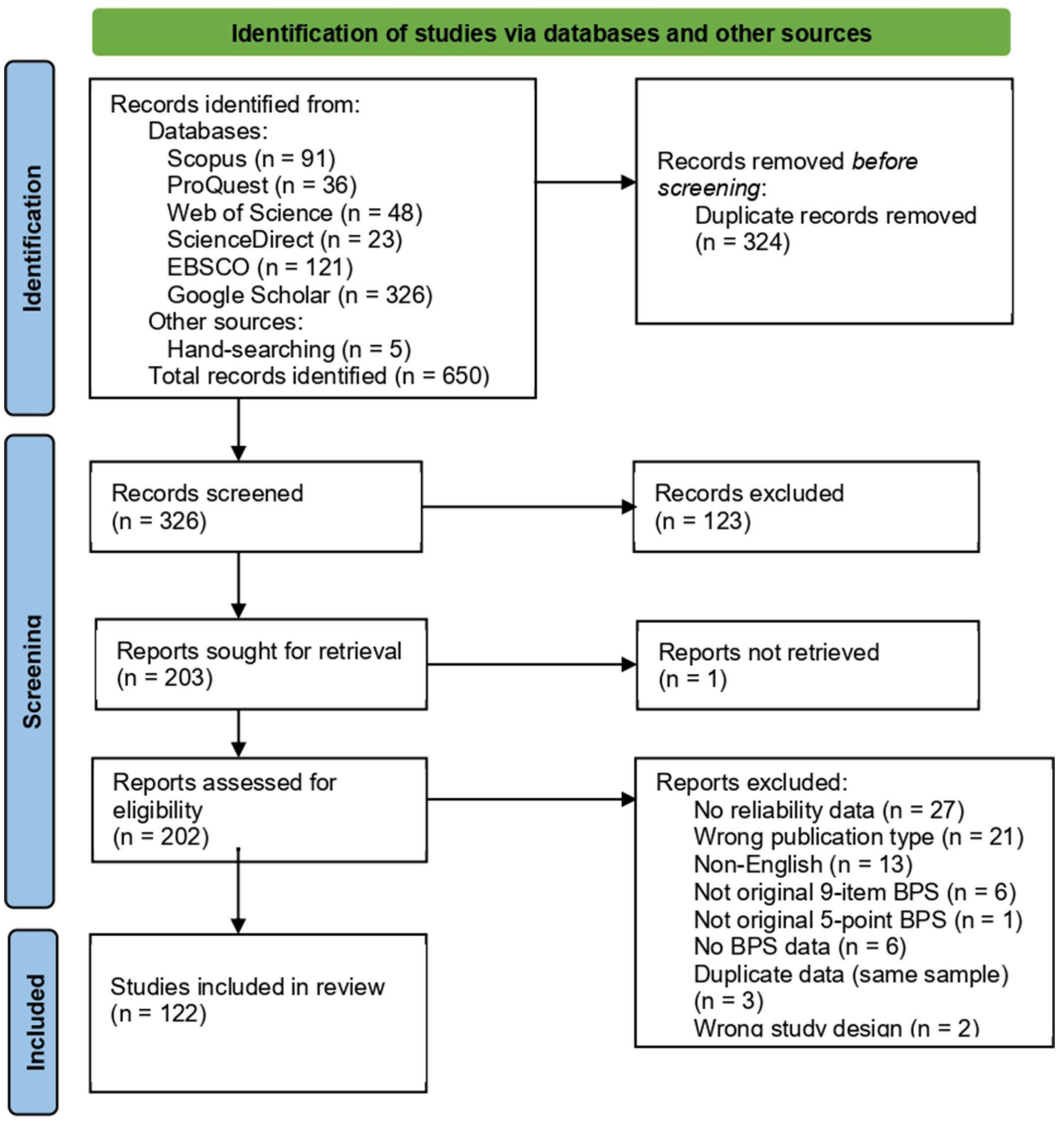
REGEMA flowchart for BPS.

Figure 1 shows the REGEMA flowchart for BPS, which summarizes the selection process of studies included in the meta-analysis. This process includes both exclusion and inclusion stages.

A total of 650 records were identified through database searching (Scopus = 91, ProQuest = 36, Web of Science = 48, ScienceDirect = 23, EBSCO = 121, Google Scholar = 326) and other sources (hand-searching = 5). After removing 324 duplicates, 326 records remained for screening. Of these, 123 were excluded based on titles and abstracts. The full texts of 202 reports were assessed for eligibility, with one report not retrieved. Among the 201 accessible reports, 76 were excluded for the following reasons: no reliability data (*n* = 27), nonacceptable publication type (*n* = 21), non-English (*n* = 13), not original 9-item 5-point BPS (*n* = 6), no BPS data (*n* = 3), duplicate data (*n* = 3), non-acceptable study design (*n* = 2), and unclear reporting of reliability (*n* = 1). Ultimately, 122 studies were included in the review. Since six of these studies were conducted on two different groups, analyses were performed with a total of 128 independent reliability coefficients. Since these subgroups were based on non-overlapping participant samples, each reliability coefficient was treated as an independent unit of analysis, consistent with recommendations for independent subgroups within studies (Borenstein et al., 2009, Chapter 23). In addition, when the same participant group was assessed at multiple time points within a study, these coefficients were not treated as independent. Instead, a single composite reliability coefficient was computed for that study by aggregating across time points, along with a corresponding mean BPS score and standard deviation. This approach follows established recommendations for handling multiple outcomes or time points based on the same participants to avoid violating independence assumptions (Borenstein et al., 2009, Chapter 24).

### Data extraction

In reliability generalization research, variability in reported reliability coefficients is commonly examined in relation to study-level methodological and sample characteristics. This variability can be attributed to the methodological characteristics including sample size (Peterson, 1994; Vassar and Bradley, 2011), publication year (Greco et al., 2018), publication type (Vassar et al., 2011; Tümtürk and Sen, 2025), mean of the scale score (Hayat, 2024; Miller et al., 2018; Tümtürk and Sen, 2025), and standard deviation of the scale score (Aguayo-Estremera et al., 2011; Miller et al., 2018), as well as participants’ demographic characteristics such as age (Pretorius and Padmanabhanunni, 2025), gender (Aguayo-Estremera et al., 2011; Vassar et al., 2011), and sample type (Peterson, 1994; Pretorius and Padmanabhanunni, 2025). In addition, contextual variables including language (Grace et al., 2018; Pretorius and Padmanabhanunni, 2025) or geographical region of the study (Aguayo-Estremera et al., 2011; Tümtürk and Sen, 2025) are frequently considered.

Consistent with prior reliability generalization studies that have examined methodological, sample-related, and contextual sources of variability, the present study operationalized these factors through a standardized data extraction procedure. For each study, the study tag (first author and year) and the publication type (e.g., peer-reviewed article and thesis) were recorded. Reported reliability coefficients were extracted, including Cronbach’s alpha and/or McDonald’s omega. Sample-related characteristics were documented, such as sample size, language of the BPS administration, country of data collection, participants’ mean age and standard deviation, sample group (e.g., university students, adolescents, and general population), and the female ratio in the sample. Descriptive statistics for the scale, including the BPS mean and standard deviation, were also extracted. Where necessary, BPS means and standard deviations reported at the total-score level were recalculated and transformed to item-level metrics to ensure consistency across studies. Specifically, some primary studies reported descriptive statistics based on the summed total BPS score, whereas others reported item-level mean scores (i.e., average per item). To ensure comparability across studies, all total-score means and standard deviations were converted to item-level means and standard deviations by dividing the total score statistics by the number of items in the BPS. This harmonization allowed all descriptive statistics to be expressed on a common item-level scale.

Study selection and coding were conducted using a multi-stage and systematic procedure. All screening and eligibility decisions were managed using the Rayyan software (Ouzzani et al., 2016). In the first stage, title and abstract screening were performed independently and in parallel by two reviewers. All records were evaluated by both reviewers and labelled in Rayyan as *include*, *exclude*, or *maybe*. Records for which the reviewers’ decisions were discordant (i.e., those marked as *conflict* in Rayyan), as well as records labelled as *maybe* by at least one reviewer, were discussed in consensus meetings involving all three authors. Final inclusion or exclusion decisions were reached by agreement, in accordance with the predefined eligibility criteria.

Studies that passed the abstract screening stage and were selected for full-text review were then examined in detail by two reviewers. For all studies deemed eligible at this stage, relevant information was coded using a standardized data extraction form. In the final stage, all data extraction sheets prepared for the included studies were independently reviewed by the third author. Any potential errors, omissions, or inconsistencies identified at this stage were re-evaluated in consultation with the other authors, and necessary corrections were made before finalizing the dataset.

This multi-stage, independent, and consensus-based review process was designed to ensure consistency and methodological rigor in the selection and coding of studies included in the meta-analysis.

### Data analysis

All analyses were conducted within a reliability generalization meta-analytic framework (Vacha-Haase, 1998; Rodríguez and Maeda, 2006) to estimate pooled internal consistency coefficients for the BPS and to examine sources of variability across studies. Both Cronbach’s alpha (*α*) and McDonald’s omega (*ω*) were included when reported. Because reliability coefficients are bounded between 0 and 1 and typically skewed, estimates were transformed prior to analysis using Bonett’s (2002) ABT variance-stabilizing transformation, which has been recommended for internal consistency coefficients such as *α* and *ω* (López-Ibáñez et al., 2024). This approach yields effect sizes with approximately normal sampling distributions and known large-sample variances. At this stage, all meta-analytic computations, including pooled reliability estimates, confidence intervals, and heterogeneity statistics, were performed using the transformed coefficients. To facilitate interpretation of the results, all pooled reliability estimates and their corresponding confidence intervals were subsequently back-transformed to the original reliability coefficient metric (Cronbach’s alpha and McDonald’s omega). Accordingly, the pooled reliability estimates and confidence intervals reported in this study are presented on the original scale familiar to readers. This approach enhances the interpretability of results obtained from statistical analyses conducted on the transformed scale in the RG study (Sánchez-Meca et al., 2013).

Random-effects models were fit using restricted maximum likelihood (REML) estimation to obtain pooled reliability estimates and between-study variance (*τ*^2^). Heterogeneity was assessed with Cochran’s *Q*, the *I*^2^ index (Higgins and Thompson, 2002), and the *H*^2^ statistic. In total, 95% prediction intervals were also calculated to describe the plausible range of reliability coefficients in future studies. Robustness was evaluated through leave-one-out influence diagnostics and examination of standardized residuals; in addition, normal Q–Q plots were inspected to assess distributional assumptions of model residuals (Viechtbauer, 2010).

Publication bias and small-study effects were examined using complementary methods. Funnel plots were inspected visually, accompanied by Egger’s regression test and Begg–Mazumdar rank correlation. Duval and Tweedie’s (2000) trim-and-fill procedure was applied as a sensitivity analysis, and precision-effect and precision-effect estimate with standard error (PET and PEESE) regressions were conducted (Stanley and Doucouliagos, 2014).

Reliability induction is a specific form of publication bias in RGMA, where the bias is introduced through the selective reporting or omission of reliability coefficients (López-Ibáñez et al., 2024). In the present study, of the 149 studies identified as using the BPS, 27 did not provide sample-specific reliability estimates: 14 did not report reliability coefficients, and 13 reported reliability values from prior studies and did not respond to author contact attempts. Accordingly, the reliability induction rate was calculated as 18.12%.

To explore heterogeneity, moderator analyses were conducted at both continuous and categorical levels. Mixed-effects meta-regressions were performed with sample characteristics (mean age, SD of age, female ratio, sample size) and scale characteristics (mean and standard deviation of BPS scores). For categorical moderators, we pre-specified the subgroup levels and applied a common-*τ*^2^ framework. For *α*, we compared effects across four moderators: (i) Region (Asia and Europe; studies from other continents or with mixed/international samples were excluded for this moderator), (ii) Scale language (Chinese, English, and Others), (iii) Sample group [adolescent, general population, and university students (undergraduate and postgraduate students combined into this category)], and (iv) Publication type (article and thesis). For *ω*, subgroup analyses were limited to the sample group (general population and university students). Levels with missing information or fewer than two independent studies were excluded *a priori* from the relevant moderator analysis to ensure stable within-group estimates (*k* ≥ 2). Other categorical moderators were not analyzed for ω due to feasibility (sparse cells for Region, Scale language) or no variability (Publication type: all articles).

Region-based analyses were restricted to Asia and Europe, as these categories represented theoretically meaningful and internally coherent groupings with sufficient numbers of studies. Studies conducted in other regions (e.g., North America and Oceania) were not combined into an “other” category because they did not share a common geographical or cultural framework that would allow for a substantively interpretable comparison. For scale language, Chinese and English versions were examined separately due to their substantial representation and distinct measurement contexts. The remaining languages were grouped under “other,” reflecting adapted versions of the BPS with small individual sample sizes that did not permit separate analysis.

All subgroup models assumed a common between-study variance (*τ*^2^) across levels. We estimated *τ*^2^ via REML and then obtained level-specific pooled effects using fixed-effect estimation on augmented variances (vi* = vi + *τ*^2^), with 95% CIs reported on the coefficient scale after back-transformation. Between-group heterogeneity was evaluated with the analog ANOVA statistic *Q*_between_ (df = group−1). Where the omnibus test was significant (*α* = 0.05), we conducted pairwise contrasts between subgroup means using Wald tests on the transformed scale and applied a Bonferroni adjustment to control family-wise error. In addition, we reported the proportion of between-study variance explained (*R*^2^) for significant moderators as $R^{2}=1-(\;\tau_{\text{within}}^{2}/\tau_{\text{between}}^{2})$ , following Borenstein et al. (2009).

As a sensitivity analysis, we additionally fitted separate random-effects models within each level of categorical moderators, allowing the between-study variance (*τ*^2^) to be estimated independently for each subgroup. These analyses revealed that *τ*^2^ values varied slightly across subgroup levels, indicating differences in residual heterogeneity. However, the pooled reliability estimates and their confidence intervals were highly consistent with those obtained under the common-*τ*^2^ specification, and the overall pattern of results remained unchanged. Accordingly, the main analyses assuming a common between-study variance are retained for presentation, with subgroup-specific *τ*^2^ estimates used to evaluate the robustness of the findings.

All analyses were carried out in R (R Core Team, 2024) using the *metafor* package (Viechtbauer, 2010). Forest plots, funnel plots, and diagnostic figures were generated with *ggplot2* and base *metafor* functions. Supplementary materials include additional diagnostic plots (trim-and-fill funnels, PET/PEESE scatterplots, Q–Q plots) and subgroup tables.

## Results

### Study characteristics

A total of 122 studies (128 reliability coefficients) were included in the reliability generalization meta-analysis of the BPS. The frequencies of categorical moderators and descriptive statistics of continuous moderators are summarized in Table 1, while Table 2 presents a summary of the coded study characteristics and moderators used in the reliability generalization meta-analysis.

**Table 1:** Frequencies of categorical moderators and descriptive statistics of continuous moderators.

Moderators		Cronbach’s alpha (f)	McDonald’s omega (f)
Categorical moderators			
Publication type	Article	118	11
	Thesis	9	–
Scale language	Chinese	49	–
	English	47	–
	Other languages	31	–
Region	Asia	84	–
	Europe	25	–
Sample group	Adolescents	12	1
	General population	33	6
	University students	76	4
	Not reported	7	–
Quantitative moderators			
	Mean	Standard deviation	
Age	22.61	9.85	
BPS score	3.14	0.83	

Due to the limited number of studies reporting McDonald’s omega, moderator analyses could not be conducted for all subgroups; therefore, frequencies are reported only for the subgroups included in the moderator analyses.

Age and BPS score statistics were computed based on 110 and 114 studies, respectively. For both continuous moderators, means and standard deviations were weighted by sample size.

**Table 2:** Summary of coded study characteristics in the reliability generalization meta-analysis of BPS.

Study tag	Publication type	Alpha	Omega	Sample size	Language of BPS	Country	Age (Mean ± SD)	Sample group	Female ratio	BPS (Mean ± SD)
Ali et al. (2021)	Article	0.782		510	Arabic	Lebanon	16.15 (3.24)	Adolescent	74.90	3.07 (0.71)
Alshammari et al. (2023)	Article	0.640		495	English	Saudi Arabia	20.89 (2.01)	University stu.	61.84	3.12 (0.76)
An and Zhang (2024)	Article	0.910		999	Chinese	China	21.16 (1.6)	General pop.	74.87	3.0 (0.8)
Andrews and Lokesh (2024)	Article	0.870		488	English	India		University stu.	50.20	2.92 (0.66)
Bernecker and Job (2019)	Article	0.890		185	German	Germany	21.73 (4.18)	University stu.	86.40	3.29 (0.58)
	Article	0.850		137	German	Germany	14.41 (0.6)	Adolescent	51.10	2.97 (0.59)
Bistricky et al. (2025)	Article	0.880		74	English	USA	25.68 (7.67)	General pop.	89.20	3.07 (0.7)
Brando-Garrido et al. (2022)	Article	0.830		177	Spanish	Spain	23.1 (4.94)	University stu.	75.71	
Broers (2014)	Thesis	0.930		153	English	USA	38.23 (13.52)	General pop.	54.20	
	Thesis	0.730		57	Dutch	Holland	21.53 (3.72)	University stu.	64.90	2.14 (0.75)
Cai et al. (2022)	Article	0.887		500	Chinese	China	19.4 (0.55)	University stu.	83.50	4.25 (0.5)
Carciofo and Cheung (2025)	Article	0.867		306	English		20.36 (4.0)	University stu.	88.88	2.8 (0.86)
Cemei et al. (2024)	Article	0.950		683	English	Pakistan	18.83 (0.19)	University stu.		3.11 (0.83)
Chen et al. (2022)	Article	0.834		1827	Chinese	China	19.07 (1.09)	University stu.	75.50	
Chen et al. (2023)	Article	0.827		576	Chinese	China	18.16 (0.73)	University stu.	44.37	3.24 (1.14)
Chung et al. (2020)	Article	0.850		106	Korean	Korea	22.7 (2.89)	General pop.	61.30	3.35 (0.66)
Cong et al. (2024)	Article	0.890		466	Chinese	China	20.18 (1.42)	University stu.	89.78	2.76 (0.83)
Correa-Iriarte et al. (2023)	Article	0.840		310	English	Spain	30.0 (10.1)	General pop.	46.80	
Cui et al. (2021)	Article	0.795		1,181	Chinese	China	18.91 (0.85)	University stu.	50.72	2.85 (0.77)
Dardara and AL-Makhalid (2021)	Article	0.610		536	English	Saudi Arabia	24.27 (5.62)	University stu.	39.60	3.77 (0.23)
Delaei-Milan (2023)	Article	0.875		133	English	Iran		University stu.		
Deng et al. (2022)	Article	0.800		913	Chinese	China	19.72 (1.24)	General pop.	54.00	3.27 (0.67)
Deng et al. (2024)	Article	0.793		2,167	Chinese	China	12.99 (1.27)	Adolescent	44.76	3.01 (0.51)
Exelmans and Bulck (2021)	Article	0.882		821	English	Belgium	45.6 (18.01)	General pop.	59.00	2.62 (0.7)
Fathy and Mandoob (2025)	Article	0.790		490	Arabic	Iraq		University stu.	73.00	2.85 (0.75)
Feng and Sun (2022)	Article	0.910		815	Chinese	China	19.53 (1.31)	University stu.	87.24	3.13 (0.71)
Feng et al. (2022)	Article	0.738		364	Chinese	China	19.48 (0.93)	University stu.	67.85	3.36 (0.74)
Flores et al. (2023)	Article	0.730		213	English			Young adults	80.28	2.99 (0.75)
Franco-Jimenez (2024)	Article		0.740	419	Spanish	Peru	21.68 (3.26)	University stu.	66.11	3.31 (0.86)
Galama (2020)	Thesis	0.920		32	Dutch	Holland	16.1 (0.81)	Adolescent	68.75	2.83 (0.82)
Geng et al. (2021)	Article	0.800		355	English	China	19.42 (1.33)	University stu.	83.10	2.75 (0.68)
Guo et al. (2020)	Article	0.787		401	Chinese	China	19.48 (0.85)	University stu.	66.08	3.18 (0.66)
Hammoudi et al. (2021)	Article	0.750		591	Arabic	Lebanon	21.13 (4.08)	University stu.	81.20	2.68 (0.73)
Hamvai et al. (2023)	Article	0.870		211	English	Hungary	22.25 (3.47)	University stu.	71.60	2.82 (0.68)
Hazumi et al. (2024)	Article	0.860	0.860	574	Japanese	Japan	44.25 (12.84)	General pop.	50.00	3.26 (0.86)
He et al. (2025)	Article	0.800		1,021	Chinese	China	18.97 (0.96)	University stu.	67.19	3.61 (0.75)
Herzog-Krzywoszanska and Krzywoszanski (2019)	Article	0.859	0.834	431	Polish	Poland	22.2 (3.23)	University stu.	88.90	3.03 (0.68)
	Article	0.862	0.839	335	Polish	Poland	38.7 (13.3)	General pop.	51.00	3.14 (0.76)
Hill et al. (2025)	Article	0.870		55	English	International	28.7 (6.5)	General pop.	25.45	3.13 (0.87)
Hou and Hu (2023)	Article	0.860		1,336	Chinese	China	19.23 (1.49)	Undergraduate & graduate stu.	65.76	3.27 (0.83)
Huang et al. (2024)	Article	0.846		1,048	Chinese	China	20.25 (2.29)	University stu.	44.20	2.79 (0.76)
Jakowski and Stork (2022)	Article	0.880		98	English	USA & Sweden	21.0 (1.7)	University stu.	62.24	3.2 (0.74)
Jakowski (2022)	Article	0.920		217	German	Germany	26.9 (7.0)	General pop.	29.00	3.14 (0.43)
Jeon et al. (2023)	Article	0.720		374	English	Korea	23.08 (2.17)	General pop.	84.50	3.24 (0.84)
Jeoung et al. (2023)	Article	0.540		60	Korean	Korea	21.33 (2.35)	Young adults	86.70	3.34 (0.83)
Jiang et al. (2024)	Article	0.910		541	Chinese	China		University stu.	94.30	2.86 (0.62)
Kadzikowska-Wrzosek (2018)	Article	0.850		304	English	Poland	28.54 (7.97)	University stu.	71.70	2.86 (0.67)
Kadzikowska-Wrzosek (2020)	Article	0.850		175	English	Poland	17.66 (0.85)	Adolescent	46.85	
Keulen (2020)	Thesis	0.900		141	English	Holland	42.3 (15.6)	General pop.	69.00	2.77 (0.96)
Krishnan and Chew (2024)	Article	0.870		221	English	Singapore	23.64 (5.72)	General pop.	63.30	3.05 (0.94)
Kroese et al. (2014)	Article	0.920		177	English	USA	39.7 (11.0)	General pop.	51.40	3.11 (0.56)
Kroese et al. (2016)	Article	0.880		2,431	English	Holland	50.7 (18.1)	General pop.	54.50	2.96 (0.6)
Kullik et al. (2025)	Article	0.930		20	English	Germany	12.9 (1.68)	Adolescent	100.00	3.38 (0.71)
Kıraç et al. (2021)	Article	0.865		768	Turkish	Türkiye		General pop.	65.90	3.23 (0.85)
Lee et al. (2025)	Article	0.824	0.817	300	Korean	Korea	17.0 (0.9)	Adolescent	50.00	2.78 (0.92)
Li et al. (2025)	Article	0.772		522	Chinese	China	29.87 (4.85)	In-service clinical nurses	88.31	3.12 (0.21)
Lin et al. (2025)	Article	0.920		1,423	Chinese	China		University stu.	80.60	2.71 (0.81)
Ling et al. (2024)	Article	0.870		763	Chinese	China	19.48 (2.06)	University stu.	64.60	
Lionnet (2023)	Thesis	0.920		327	English	New Zealand	20.93 (6.34)	University stu.	80.70	3.22 (0.86)
Liu et al. (2024)	Article	0.853		4,196	English	China	29.17 (0.14)	General pop.	42.28	3.48 (0.72)
Liu et al. (2025)	Article	0.840		990	Chinese	China	23.06 (4.21)	University stu.	46.06	2.89 (0.8)
Luo et al. (2024)	Article	0.890	0.892	252	English	China	20.32 (1.47)	University stu.	100.00	2.52 (0.32)
Ma et al. (2022)	Article	0.820		1,550	Chinese	China	19.3 (0.98)	University stu.	69.29	3.22 (0.77)
Mao et al. (2022)	Article	0.850		3,687	Chinese	China	16.17 (2.42)	General pop.	57.23	3.17 (0.81)
Meng and Xuan (2023)	Article	0.868		707	Chinese	China		University stu.	70.16	3.25 (0.8)
Meng et al. (2021)	Article	0.867		267	Chinese	China		University stu.	67.41	
	Article	0.863		361	Chinese	China		University stu.	73.68	3.38 (0.83)
Meng et al. (2022)	Article	0.831		552	Chinese	China	19.22 (0.64)	University stu.	62.86	3.01 (0.85)
Meng et al. (2024)	Article	0.982		3,599	Chinese	China	19.12 (1.05)	University stu.	44.80	2.7 (0.8)
Meng et al. (2023)	Article	0.855		583	Chinese	China	20.02 (1.85)	University stu.	71.35	2.98 (0.36)
Miyagawa et al. (2024)	Article	0.900	0.920	252	Japanese	Japan	39.36 (9.27)	General pop.	55.60	2.97 (0.83)
	Article	0.900	0.920	630	Japanese	Japan	37.69 (12.82)	General pop.	57.20	
Mu et al. (2023)	Article	0.860		271	Chinese	China	21.5 (2.8)	University stu.	58.30	2.86 (0.73)
Nauts et al. (2016)	Article	0.910		234	English	USA	37.1 (13.48)	General pop.	42.31	2.7 (0.78)
Okay et al. (2022)	Article	0.910		317	Turkish	Türkiye	21.78 (3.94)	General pop.	78.86	2.97 (0.77)
Oliveira et al. (2022)	Article	0.900		560	Portuguese	Portugal	29.85 (12.83)	General pop.	74.50	3.2 (0.88)
Oliveira et al. (2025)	Article	0.900	0.900	653	Portuguese	Portugal	29.8 (12.45)	General pop.	74.70	2.66 (0.72)
Pillion (2023)	Thesis	0.720		711	English	Australia	15.1 (1.2)	Adolescent	47.30	2.8 (0.41)
Pu et al. (2022)	Article	0.850		121	English	Singapore	15.9 (1.14)	Adolescent	54.55	3.08 (0.75)
Pu et al. (2025)	Article	0.850		119	English	Singapore	22.66 (1.67)	University stu.	53.78	2.25 (0.77)
Qin et al. (2025)	Article	0.873		769	English		20.89 (1.63)	University stu.	75.90	3.38 (1.24)
Rahayu and Caninsti (2024)	Article	0.678		192	Indonesian	Indonesia		Adolescent	75.00	3.22 (0.84)
Rapoport et al. (2025)	Article	0.910		262	German	Germany	35.35 (14.05)	General pop.	66.41	3.03 (0.77)
Rasouli et al. (2025)	Article	0.840		433	Persian	Iran	22.57 (3.52)	General pop.	55.70	3.23 (0.89)
Rehman et al. (2023)	Article	0.920	0.900	241	English	Pakistan	29.72 (9.3)	General pop.	87.5	2.79 (0.65)
Saed et al. (2019)	Article	0.820		433	English	Iran		University stu.	55.66	2.91 (0.8)
Santos (2020)	Thesis	0.900		446	Portuguese	Portugal	23.7 (5.49)	University stu.	70.00	3.11 (0.85)
Sezer et al. (2025)	Article	0.880		336	English	UK	43.11 (11.41)	General pop.	56.00	3.19 (0.62)
Shao et al. (2024)	Article	0.800		453	Chinese	China	21.21 (1.59)	University stu.	44.80	
Shi et al. (2024)	Article	0.770		737	Chinese	China	20.05 (1.38)	University stu.	80.87	3.08 (0.61)
Shoukat et al. (2025)	Article	0.780		300	English	Pakistan		University stu.	50.00	3.25 (1.69)
Shukla and Andrade (2023)	Article	0.780		560	English	India	19.8 (1.9)	Undergraduate & graduate stu.	57.50	3.12 (0.82)
Sirois et al. (2019)	Article	0.890		134	English	UK	30.22 (13.5)	General pop.	77.40	3.07 (0.76)
	Article	0.900		646	English	UK	30.74 (12.2)	General pop.	68.90	
Song et al. (2024)	Article	0.824	0.817	300	Korean	Korea	17.0 (0.9)	University stu.	50.00	2.68 (0.68)
Teoh and Wong (2023)	Article	0.870		220	English	Australia	20.34 (2.86)	University stu.	67.73	3.49 (0.76)
Teoh et al. (2021)	Article	0.830		270	English	Singapore	22.39 (5.41)	University stu.	73.33	2.74 (0.76)
Trost and Hast (2024)	Article	0.890		418	German	Germany	23.3 (3.0)	Young adults	83.60	3.62 (0.64)
Tu et al. (2023)	Article	0.790		910	Chinese	China	20.14 (3.48)	University stu.	60.00	3.02 (0.39)
Türkarslan et al. (2020)	Article	0.910		229	Turkish	Türkiye	27.82 (10.81)	General pop.	68.12	3.1 (0.74)
Uygur and Bahar (2023a)	Article	0.610		497	Turkish	Türkiye	20.41 (1.83)	University stu.	72.80	3.42 (0.77)
Uygur and Bahar (2023b)	Article	0.760		553	Turkish	Türkiye	20.55 (2.17)	University stu.	69.60	2.8 (0.8)
Wang et al. (2024)	Article	0.840		54	Chinese	China	19.8 (0.6)	University stu.	92.60	
Wang et al. (2025)	Article	0.876		935	Chinese	China		University stu.	53.50	4.17 (1.43)
Washof (2023)	Thesis	0.750		149	Dutch	Holland	38.8 (13.3)	General pop.	53.69	3.25 (0.73)
Xu et al. (2024)	Article	0.890		855	Chinese	China	21.16 (1.83)	University stu.	46.20	
Yang et al. (2023)	Article	0.804		1,217	Chinese	China	20.3 (2.2)	University stu.	42.65	3.11 (0.98)
Yang et al. (2024)	Article	0.850		2044	English	China		University stu.	63.11	3.71 (0.3)
Yasin et al. (2024)	Article	0.740		182	English	Pakistan	21.98 (2.17)	University stu.	100.00	3.01 (0.41)
Yinn et al. (2024)	Article	0.852		108	English	Malaysia	22.27 (1.89)	Young adults	57.40	3.1 (0.8)
You et al. (2020)	Article	0.880		1,104	Chinese	China	20.2 (1.43)	University stu.	63.00	2.94 (0.93)
You et al. (2023)	Article	0.880		1,103	Chinese	China	20.17 (1.43)	University stu.	63.00	3.02 (0.66)
Yu et al. (2025)	Article	0.872		356	Chinese	China		University stu.	50.60	2.46 (0.63)
Yuan et al. (2024)	Article	0.840		464	English	China	21.7 (3.1)	University stu.	65.50	3.15 (0.87)
Zhang and Shi (2025)	Article	0.870		2052	Chinese	China	20.0 (1.53)	University stu.	68.40	2.91 (0.63)
Zhang and Wu (2020)	Article	0.790		427	English	China	19.36 (1.06)	University stu.	66.00	3.13 (1.01)
Zhang (2024)	Thesis	0.860		105	English	International	23.9 (4.07)	University stu.	73.38	
Zhang C. et al. (2023)	Article	0.805		318	Chinese	China	16.92 (0.67)	Adolescent	64.20	3.2 (0.56)
Zhang M. X. et al. (2023)	Article	0.813		698	Chinese	China	20.15 (1.77)	University stu.	33.38	3.41 (0.77)
Zhang et al. (2024)	Article	0.830		3,539	Chinese	China	15.6 (2.9)	Elementary to college stu.	59.80	3.36 (0.87)
Zhang et al. (2025)	Article	0.917		403	English	Pakistan	23.42 (4.2)	University stu.	58.31	3.28 (0.91)
Zhao et al. (2024)	Article	0.874		288	Chinese	China	20.89 (2.27)	Undergraduate & graduate stu.	68.40	2.98 (0.75)
Zhuo (2024)	Article	0.860		474	Chinese	China		University stu.	53.00	3.23 (0.84)
Zhou et al. (2025)	Article	0.922		6,543	Chinese	China		University stu.	64.70	3.19 (0.84)
Zhu et al. (2020)	Article	0.795		391	Chinese	China	19.48 (0.86)	University stu.	66.75	3.34 (0.81)
Zhu et al. (2022)	Article	0.760		2,822	Chinese	China	19.77 (1.41)	University stu.	71.40	3.55 (0.64)
Zhu et al. (2023)	Article	0.780		668	Chinese	China	20.36 (1.69)	University stu.	64.97	2.92 (0.66)
Zhu et al. (2024)	Article	0.830		665	Chinese	China	13.72 (1.64)	Adolescent	49.77	3.29 (0.87)

When the study tag includes “a” and “b” within the year (e.g., Uygur and Bahar, 2023a,b), this denotes two distinct studies by the same author in the same year. stu., students; pop., population. Empty cells denote missing information in the original study. For consistency, BPS mean and SD values were recalculated and transformed to item-level metrics when reported as total scores.

The majority were journal articles, with a smaller number of theses. Sample sizes varied widely, ranging from very small groups of fewer than 30 participants to large-scale studies with several thousand respondents. Studies represented diverse geographical regions and languages, including Chinese, English, Arabic, Turkish, German, Spanish, Portuguese, and others, reflecting broad international use of the BPS.

Participants encompassed a variety of groups, most commonly university students, but also adolescents, general population samples, and young adults. The average age across samples ranged from early adolescence to middle adulthood, with female participation rates differing substantially across studies.

Internal consistency estimates (Cronbach’s alpha and, where available, McDonald’s omega) showed considerable variability across studies. In several cases, more than one coefficient was reported within a single publication due to analyses conducted on multiple groups. When the same study tag appears with suffixes “a” and “b,” this denotes distinct groups within the same study; when tags include “_1” and “_2,” this indicates separate studies conducted by the same author in the same year. Empty cells reflect missing information in the original reports.

### Bps alpha

#### Publication and reporting biases (*α*)

**Figure 2:**
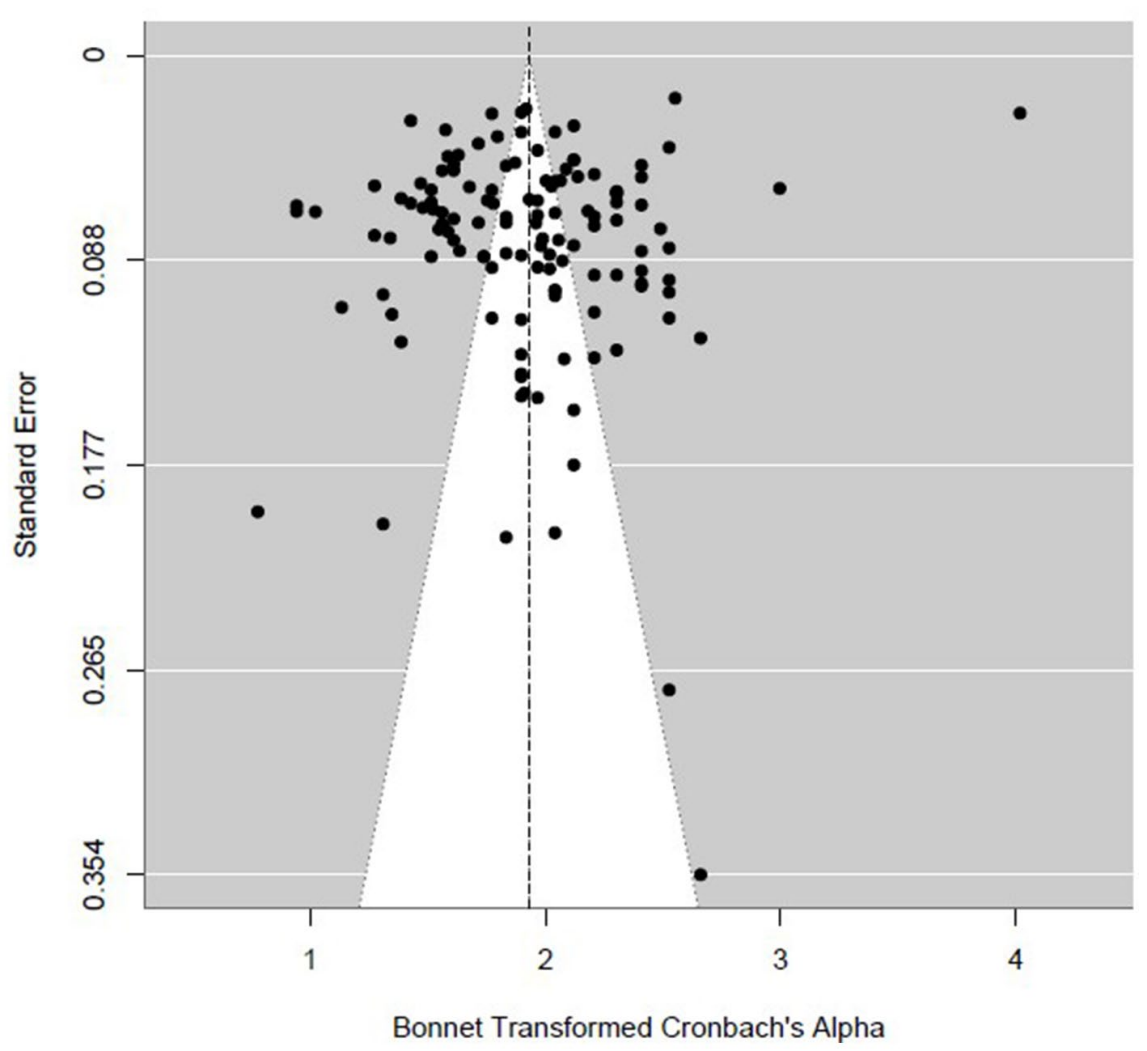
Funnel plot of Cronbach’s alpha (Bonett-transformed) for the BPS.

Publication bias was examined using multiple, complementary diagnostics based on Bonett’s ABT transformation of Cronbach’s alpha. Visual inspection of the funnel plot (Figure 2) shows the distribution of individual studies around the pooled estimate, plotted against the standard error, allowing assessment of potential small-study effects. Visual inspection was paired with formal tests, which did not indicate clear asymmetry: Egger’s regression was non-significant (*z* = 0.268, *p* = 0.789), and Begg–Mazumdar’s rank correlation was also non-significant (Kendall’s *τ* = 0.110, *p* = 0.068). As a sensitivity check, Duval and Tweedie’s trim-and-fill procedure imputed *k*₀ = 20 potentially missing studies and yielded a downward-adjusted pooled reliability of *α* = 0.836 [95% CI (0.822, 0.850)], compared with the original REML estimate of *α* = 0.855 [95% CI (0.843, 0.865)]; this corresponds to an absolute change of −0.019 (≈ −2.16%), suggesting that possible unpublished (or published but that not report the empirical reliability) studies would have only a modest impact on the pooled estimate.

To further probe small-study effects, PET and PEESE meta-regressions produced bias-adjusted intercepts of *α* = 0.8518 and *α* = 0.8515, respectively, which closely align with the original pooled estimate, suggesting minimal impact of small-study/publication bias on the central estimate. Taken together, the evidence is mixed. That is, trim-and-fill indicates possible missing, less precise studies that would slightly lower the pooled reliability, whereas Egger, Begg-Mazumdar, PET, and PEESE provide little support for material small-study bias. The pooled reliability estimate is therefore interpreted as robust, with all diagnostics reported for transparency (see Supplementary Figures S1–S3: trim-and-fill funnel, PET, and PEESE scatterplots).

#### Mean reliability and heterogeneity (*α*)

A random-effects meta-analysis was conducted on 127 (one study only reported omega) independent samples using Bonett’s ABT transformation of Cronbach’s alpha (REML estimator). The pooled effect on the ABT scale^1^ was 1.9286 [SE = 0.0388, *z* = 49.71, *p* < 0.0001; 95% CI (1.8525, 2.0046)], which back-translates to a mean reliability of *α* = 0.8546 [95% CI (0.8432, 0.8653)]. Between-study heterogeneity was very large: *τ*^2^ = 0.1827 (SE = 0.0240), *τ* = 0.4274, *I*^2^ = 98.24%, *H*^2^ = 56.88; Cochran’s *Q*_(126)_ = 11,892.77, *p* < 0.0001. All heterogeneity statistics (*τ*^2^ and *τ*) are reported on the transformed (ABT) scale. Reflecting this heterogeneity, the 95% prediction interval on the alpha scale was wide (0.6629–0.9373), indicating that future studies conducted under similar conditions may plausibly yield reliability estimates across this range. The dispersion of study-specific estimates is visualized in the forest plot (see Supplementary Figure S4), which illustrates both the concentration of effects around the pooled estimate of the mean and the presence of studies with lower and higher reliability.

Leave-one-out diagnostics did not reveal undue influence by any single study. Across the most influential omissions identified, the back-transformed pooled *α* remained tightly bounded (approximately 0.852–0.856), while heterogeneity indices stayed high (e.g., *I*^2^ ≈ 97.7–98.2%; *τ*^2^ ≈ 0.146–0.181). A Q–Q plot of standardized residuals (see Supplementary Figure S5) further indicated approximate normality, with most studies following the theoretical quantile line reasonably well and only modest deviations in the tails. This pattern supports the robustness of the central estimate while reflecting the very high heterogeneity observed across studies. Taken together, the central estimate of reliability is stable, but the magnitude of heterogeneity suggests that moderator analyses are warranted to explain systematic variability across studies.

#### Meta regressions for continuous moderator variables (*α*)

Mixed-effects meta-regressions were conducted to examine whether sample characteristics and scale scores accounted for heterogeneity in Cronbach’s alpha coefficients of the BPS (Table 3). Mean age was positively associated with reliability estimates, *b* = 0.016, 95% CI [0.005, 0.027], *p* = 0.005, explaining 6.87% of the heterogeneity. Similarly, age variability (sd of age) was a significant positive predictor, *b* = 0.030, 95% CI [0.012, 0.048], *p* = 0.001, accounting for 8.75% of the heterogeneity (see Supplementary Figures S6, S7). That is, studies with older samples and greater age variability tend to report higher reliability estimates for the BPS scores. By contrast, the proportion of women in the sample was unrelated to reliability (*p* = 0.935). With respect to sample size, the raw n specification showed a marginal trend, *b* ≈ 0.000, 95% CI [−0.000, 0.000], *p* = 0.072, explaining 2.07% of heterogeneity, whereas the log-transformed *N* was clearly nonsignificant (*p* = 0.619). For scale score moderators, neither the mean BPS score (*b* = −0.200, 95% CI [−0.470, 0.070], *p* = 0.144) nor the SD of BPS scores (*b* = 0.197, 95% CI [−0.231, 0.625], *p* = 0.365) significantly predicted reliability. Both explained negligible portions of heterogeneity (≤1.3%). Overall, while older average age and greater age variability of participants were associated with higher reliability, these effects were small. Moreover, the persistence of very high I^2^ values should be interpreted cautiously, as *I*^2^ is a relative measure of heterogeneity and may remain inflated in meta-analyses with generally large sample sizes and very small sampling variances, even when the inclusion of moderators leads to only modest reductions in the true between-study variance (*τ*^2^).

**Table 3:** Mixed-effects meta-regression results for continuous moderators of BPS (*α*).

Moderator	*k*	*τ* ^2^	*I*^2^ (%)	*R*^2^ (%)	Estimate (*b*)	95% CI	*p*
Mean age	110	0.182	98.10	6.87	0.016	0.005, 0.027	0.005*
SD age	109	0.175	98.04	8.75	0.030	0.012, 0.048	0.001*
Women (%)	125	0.177	98.19	0.00	0.000	−0.005, 0.005	0.935
Sample size (*n*)	127	0.179	98.13	2.07	0.000	−0.000, 0.000	0.072
Sample size (log *n*)	127	0.184	98.21	0.00	0.020	−0.059, 0.100	0.619
Mean BPS score	113	0.194	98.39	1.27	−0.200	−0.470, 0.070	0.144
sd BPS score	113	0.197	98.42	0.00	0.197	−0.231, 0.625	0.365

*p < 0.05; *k*: number of alphas included.

#### Subgroup analyses for categorical moderator variables (*α*)

Subgroup analyses (Table 4) using a common-*τ*^2^ model showed a significant difference by Region [*Q*_(1)_ = 5.776, *p* = 0.016], with Europe exhibiting higher reliability (*α* ≈ 0.877) than Asia (*α* ≈ 0.845). That is, studies conducted in Europe tend to report more reliable BPS scores than studies conducted in Asia. Scale language showed no differences [*Q*_(2)_ = 0.539, *p* = 0.764]. Sample group was significant [*Q*_(2)_ = 10.742, *p* = 0.005]; pairwise tests (Bonferroni) indicated that the general population had higher reliability than university students (*α* ≈ 0.882 vs. 0.849; *p_adj* = 0.016) and adolescents (*α* ≈ 0.882 vs. 0.825; *p_adj* = 0.018), whereas adolescents and university students did not differ (*α* ≈ 0.825 vs. 0.849; *p_adj* = 0.760) (see Supplementary Table S1). In other words, BPS scores appear to be more reliable in studies based on general population samples than in studies focusing on university students or adolescents. Publication type showed no difference [*Q*_(1)_ = 0.421, *p* = 0.516]. The moderators explained ≈4.1% (Region) and ≈7.5% (Sample group) of between-study variance (*R*^2^; see Supplementary Table S2).

**Table 4:** Subgroup analyses for categorical moderators (*α*).

Moderator	Category	*k*	Alpha [CI]	*Z*	*Q*	df	*p*
Region	Asia	84	0.845 [0.830–0.858]	40.903*	5.776*	1	0.016
	Europe	25	0.877 [0.855–0.896]	24.499*			
Scale language	Chinese	49	0.854 [0.835–0.871]	30.954*	0.539	2	0.764
	English	47	0.859 [0.840–0.876]	30.329*			
	Others	31	0.849 [0.823–0.870]	23.721*			
Sample group	Adolescent	12	0.825 [0.777–0.863]	14.088*	10.742*	2	0.005
	General population	33	0.882 [0.864–0.897]	29.439*			
	University students	76	0.849 [0.835–0.863]	39.931*			
Publication type	Article	118	0.854 [0.842–0.865]	47.748*	0.421	1	0.516
	Thesis	9	0.868 [0.822–0.902]	13.445*			

*p < 0.05; *k:* number of alphas included.

Alpha coefficients were pooled on the alpha-based transformation (ABT) scale. The *Z* statistic tests whether the pooled alpha estimate for each subgroup differs significantly from zero. The *Q* statistic, along with its degrees of freedom (df) and *p*-value, represents the between-groups omnibus test assessing whether pooled alpha coefficients differ significantly across categories of the moderator (i.e., a meta-analytic analogue of ANOVA).

### Bps omega

#### Publication and reporting biases (*ω*)

**Figure 3:**
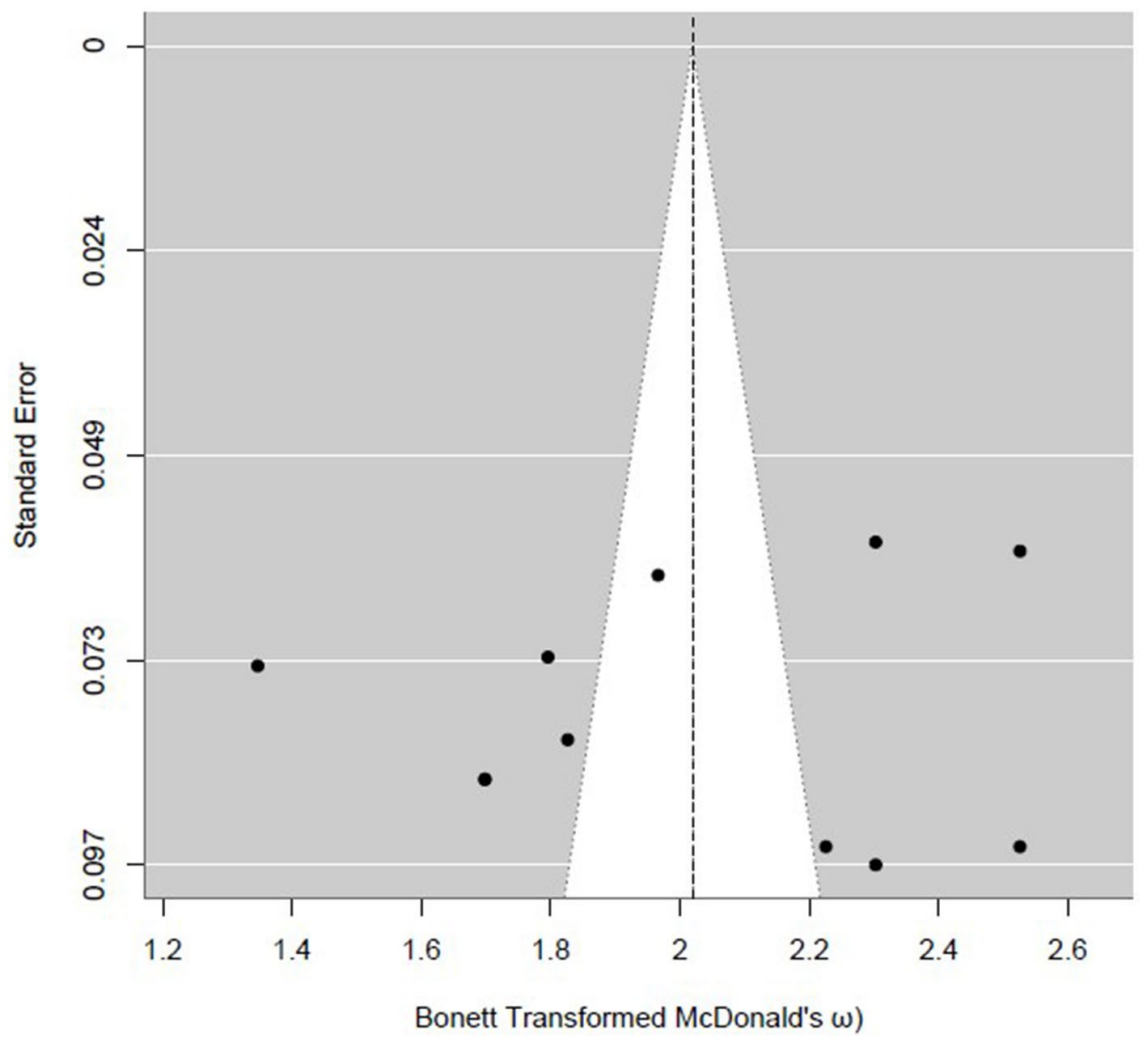
Funnel plot of McDonald’s omega (Bonett-transformed) for the BPS.

Publication bias was assessed using multiple, complementary diagnostics on the Bonett-transformed scale. Formal tests did not indicate asymmetry: Egger’s regression was non-significant (*z* = 0.0496, *p* = 0.9604), and Begg–Mazumdar’s rank correlation was also non-significant (Kendall’s *τ* = −0.0561, *p* = 0.8137). Visual inspection of the funnel plot suggested symmetry (Figure 3).

As a sensitivity check, Duval and Tweedie’s trim-and-fill procedure imputed *k*₀ = 0 studies and left the pooled estimate unchanged at *ω* = 0.867 [95% CI (0.833, 0.894)]. PET and PEESE meta-regressions yielded bias-adjusted intercepts close to the pooled estimate (PET *ω* = 0.8625; PEESE *ω* = 0.8561). Taken together, these indicators provide little evidence of material small-study/publication bias, and the central reliability estimate appears robust (see Supplementary Figures S8–S10: trim-and-fill funnel, PET, and PEESE scatterplots).

#### Mean reliability and heterogeneity (*ω*)

**Figure 4:**
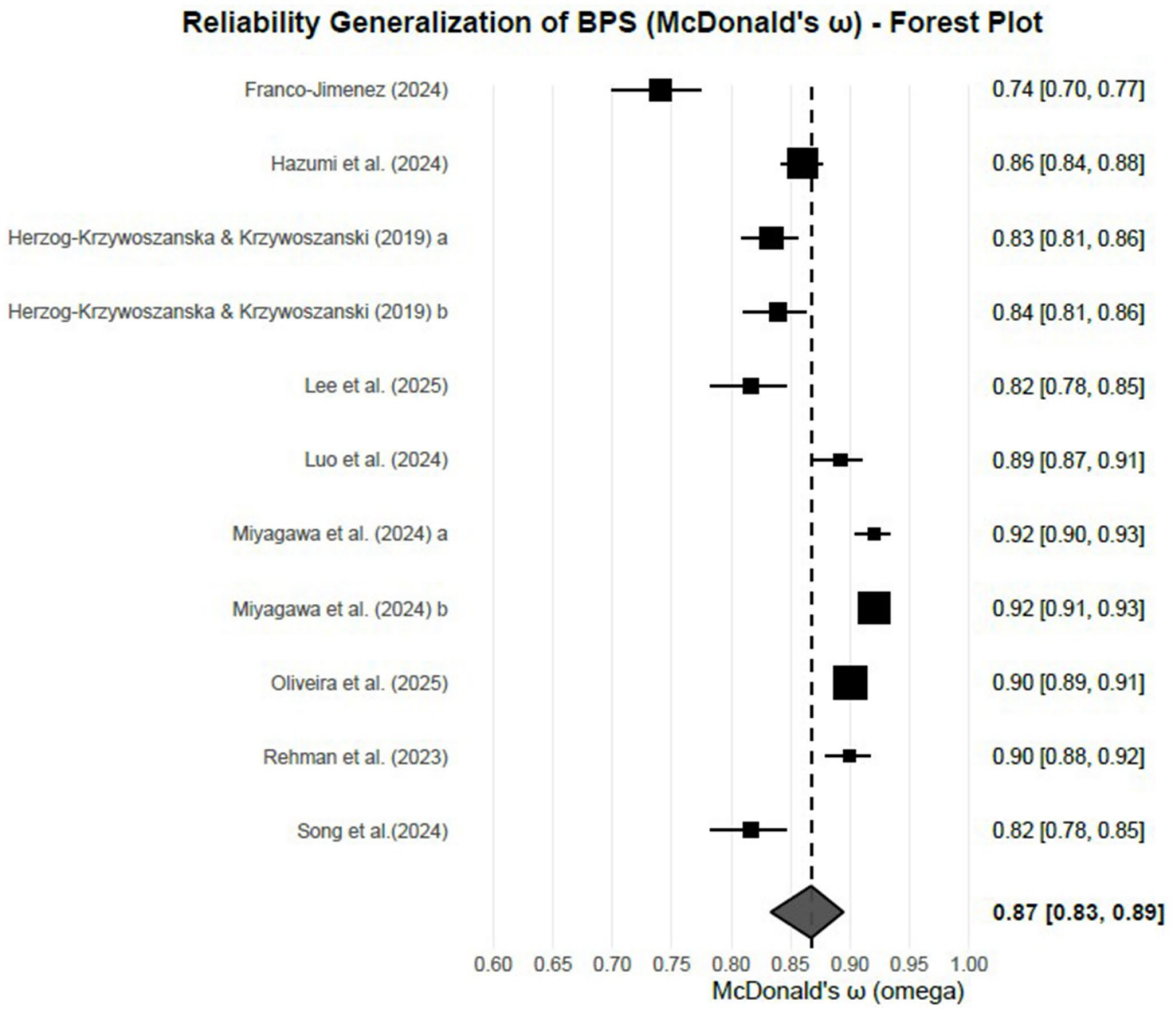
Forest plot of McDonald’s *ω* for the BPS.

A random-effects meta-analysis on *k* = 11 independent samples yielded a pooled effect of 2.019 on the ABT transformed scale [SE = 0.116, *z* = 17.48, *p* < 0.0001; 95% CI (1.793, 2.246)]. Back-transformed to McDonald’s omega, the mean reliability was *ω* = 0.867 with 95% CI [0.834, 0.894]. Between-study heterogeneity was substantial: *τ*^2^ = 0.140 (SE = 0.066), *τ* = 0.375, *I*^2^ = 96.07%, *H*^2^ = 25.41; Cochran’s *Q*_(10)_ = 262.60, *p* < 0.0001. Consistent with this dispersion, the 95% prediction interval on the omega scale was [0.714, 0.938], indicating that future studies conducted under similar conditions may plausibly yield reliability estimates across this range. Study-level estimates and their confidence intervals are displayed in the forest plot (Figure 4). Individual study estimates with 95% confidence intervals are shown as squares and horizontal lines, respectively. The size of the square reflects the study’s weight, and the diamond represents the pooled reliability estimate with its confidence interval.

Leave-one-out analyses did not indicate undue influence by any single study. Across the most influential omissions, heterogeneity remained high (*I*^2^ ≈ 94.56–96.46%; *τ*^2^ ≈ 0.101–0.152), and the pooled effect on the transformed scale remained within a narrow band, implying a stable central estimate despite notable between-study variability. A Q–Q plot of standardized residuals (see Supplementary Figure S11) further indicated approximate normality, with most studies following the theoretical quantile line and only modest deviations at the extremes. This supports the robustness of the central estimate while highlighting the very high heterogeneity observed across studies.

#### Meta regressions for continuous moderator variables (*ω*)

Mixed-effects meta-regressions were conducted to examine whether sample characteristics and scale scores accounted for heterogeneity in McDonald’s *ω* coefficients of the BPS (Table 5). None of the continuous moderators reached statistical significance (all *p* ≥ 0.086). For age-related moderators, mean age showed a non-significant positive association, *b* = 0.020, 95% CI [−0.005, 0.045], *p* = 0.099, and age variability (sd of age) was likewise non-significant, *b* = 0.039, 95% CI [−0.007, 0.085], *p* = 0.086. The proportion of women was unrelated to reliability, *b* = 0.004, 95% CI [−0.011, 0.020], *p* = 0.546. Neither sample size in raw units [*b* ≈ 0.000, 95% CI (−0.001, 0.002), *p* = 0.656] nor on the log scale [*b* = 0.042, 95% CI (−0.724, 0.808), *p* = 0.903] predicted *ω*. For scale-score moderators, mean BPS [*b* = −0.612, 95% CI (−1.587, 0.363), *p* = 0.186] and sd of BPS [*b* = −0.738, 95% CI (−2.385, 0.909), *p* = 0.332] were also non-significant. Overall, residual heterogeneity remained very high (*I*^2^ ≈ 95–96%), indicating that most between-study variability in ω was not explained by these moderators with the available *k* (10–11 studies per model).

**Table 5:** Mixed-effects meta-regression results for continuous moderators of BPS (*ω*).

Moderator	*k*	*τ* ^2^	*I*^2^ (%)	*R*^2^ (%)	Estimate (*b*)	95% CI	*p*
Mean age	11	0.112	95.09	20.00	0.020	−0.005, 0.045	0.099
SD age	11	0.108	94.92	22.75	0.039	−0.007, 0.085	0.086
Women (%)	11	0.150	96.33	0.00	0.004	−0.011, 0.020	0.546
Sample size (*n*)	11	0.153	96.30	0.00	0.000	−0.001, 0.002	0.656
Sample size (log *n*)	11	0.156	96.41	0.00	0.042	−0.724, 0.808	0.903
Mean BPS score	10	0.109	94.57	11.88	−0.612	−1.587, 0.363	0.186
SD BPS score	10	0.123	95.35	1.01	−0.738	−2.385, 0.909	0.332

*p < 0.05; *k*: number of omegas included.

### Subgroup analyses for categorical moderator variables (*ω*)

Subgroup analyses using a common-*τ*^2^ model compared university students and the general population (Table 6). The between-groups test was significant, *Q*_(1)_ = 5.445, *p* = 0.020, with higher reliability in general population samples [*ω* = 0.894, 95% CI (0.863, 0.917); *k* = 6] than in university student samples [*ω* = 0.828, 95% CI (0.766, 0.874); *k* = 4]. The sample-group moderator explained ≈34.7% of the between-study variance (*R*^2^; see Supplementary Table S3). Other categorical moderators were not analyzed for *ω* due to feasibility (sparse cells for Region, Scale language) or no variability (Publication type: all articles).

**Table 6:** Subgroup analysis for sample group (*ω*).

Sample group	*k*	*ω* [95% CI]	*Z*	*Q*	df	*p*
General population	6	0.894 [0.863–0.917]	17.345*	5.445*	1	0.020
University students	4	0.828 [0.766–0.874]	11.097*			

*p < 0.05; *k*: number of omegas included.

*Z*-statistic tests pooled effects on the ABT scale. *Q*/df/*p* are the between-groups omnibus tests (analog ANOVA).

## Discussion

Reliability is crucial in psychological assessment because it ensures the consistency and accuracy of the data collected. Unreliable data can compromise the validity of research findings and lead to incorrect conclusions. Using the REGEMA framework, the present study aimed to evaluate the reliability of the Bedtime Procrastination Scale (BPS) across diverse cultural, linguistic, and sample characteristics. Accordingly, reliability generalization meta-analyses were conducted to estimate the pooled reliability of the BPS using two internal consistency coefficients—Cronbach’s alpha and McDonald’s omega—and to investigate potential moderator variables that may account for variability in reliability estimates across individual studies. The results indicated that the pooled reliability estimates were 0.855 for Cronbach’s alpha and 0.867 for McDonald’s omega. It should be noted that the pooled McDonald’s omega estimate was based on a smaller number of studies. The pooled reliability estimates were higher than the commonly accepted threshold of 0.70 (Cohen et al., 2022; George and Mallery, 2020; Nunnally and Bernstein, 1994). While this cut-off is considered sufficient for studies focusing on predictive or construct validity (Nunnally and Bernstein, 1994), higher thresholds of 0.90 or 0.95 are recommended in contexts involving high risk or critical decision-making (Cohen et al., 2022; Nunnally and Bernstein, 1994). From a construct validity perspective, the pooled reliability estimates obtained in this study can therefore be considered acceptable.

In addition, 95% prediction intervals were estimated for both Cronbach’s alpha and McDonald’s omega (for Cronbach’s alpha: 0.6629–0.9373; for McDonald’s omega: 0.714–0.938). Prediction intervals provide an estimate of the range within which reliability coefficients of future studies are expected to fall (Higgins et al., 2009; IntHout et al., 2016). The relatively wide prediction intervals observed in this study indicate that caution is warranted, particularly in situations involving high-stakes or critical decisions.

Identifying sources affecting the homogeneity of reliability is another key point of the study. After estimating pooled reliability, the homogeneity of reliability coefficients was assessed using Cochran’s *Q*, the *I*^2^ index (Higgins and Thompson, 2002), and the H^2^ statistic. The results showed that in the analysis of internal consistency coefficients such as Cronbach’s alpha and McDonald’s omega, there was a significant degree of variability between studies, commonly referred to as inter-study heterogeneity. Moderator analyses revealed that various characteristics were statistically significant predictors of variability in reliability estimates. However, the heterogeneity explained by these moderators was generally found to be low. This may be partly due to the large number of studies included in the analysis, whereby even small differences in a large sample can become statistically significant (Borenstein et al., 2009). Furthermore, it highlights the need for careful interpretation of statistical significance and that it should not be equated with practical importance.

In this study, mean age, standard deviation of age, proportion of female participants, sample size, mean BPS scores, and standard deviation of BPS scores were included as continuous moderators, and analyses were conducted to account for the observed heterogeneity in Cronbach’s alpha and McDonald’s omega coefficients. Meta-regression results for Cronbach’s alpha revealed that both mean age and age variability (standard deviation of age) were significantly associated with reliability, indicating that higher average age and greater dispersion in participant ages corresponded to increased Cronbach’s alpha values. Supporting this perspective, Schipke and Freund (2012) reported that samples consisting solely of adults or solely of older individuals negatively affected reliability, whereas larger sample sizes tended to have a positive influence. By contrast, no significant associations were observed for female proportion, sample size, mean BPS score, or standard deviation of BPS score, which showed that gender distribution, sample size, and score averages were not statistically significant predictors of heterogeneity of reliability. The fact that the effect of the female ratio on reliability coefficients is statistically insignificant indicates that similar levels of reliability evidence have been achieved in terms of the gender variable. Indeed, Franco-Jimenez (2024), in their study on the Bedtime Procrastination Scale, examined measurement invariance by gender and found that the scale provided measurement invariance across genders. This finding provides evidence for the construct validity of the scale across genders. Therefore, it can be said that the finding that reliability does not differ by gender is consistent with previous findings regarding construct validity from a psychometric perspective. Sample size was also a non-significant moderator. The content of BPS may facilitate stable reliability estimates, reducing the need for large samples to achieve adequate measurement precision. Notably, in the original development study of the instrument, data collected from 177 participants already provided sufficient evidence for the psychometric properties. For McDonald’s omega coefficients, however, none of the continuous moderator variables, including mean age and age variability, were found to be significant. This may be attributable to the relatively small number of studies reporting omega, which reduced the power of the significance test of the moderators in participant age across samples. Indeed, the studies included in the omega analyses predominantly encompassed participants with a narrower age range compared to those in the Cronbach’s alpha analyses.

Subgroup analyses of Cronbach’s alpha for categorical moderator variables revealed significant effects for region and sample group, whereas scale language and publication type were not significant predictors of variability in reliability coefficients. With respect to region, studies conducted in Europe yielded higher reliability estimates compared to those conducted in Asia. For the categorical moderator variable of sample group, which distinguished between adolescents, university students, and the general population, results indicated that the general population group demonstrated significantly higher reliability compared to both adolescents and university students. However, no significant differences were observed between the adolescent and university student samples. Bruna et al. (2018) associated the high heterogeneity between samples with the variability of reliability. Despite the presence of significant categorical moderators, neither scale language nor publication type emerged as a significant moderator. Non-significance of the language can be explained by the psychometric properties of the BPS. The instrument is a brief, unidimensional measure consisting of nine items that are conceptually straightforward and terminologically simple. These features likely facilitate practical adaptation across languages while maintaining the integrity of the factor structure, thereby preserving response consistency across translations. Moreover, although the original validation study was conducted with a relatively small sample, construct validity was nonetheless supported, further suggesting that the scale’s structure is robust and easily replicable across different linguistic and cultural contexts. For McDonald’s omega, categorical moderator analyses were limited to sample type (university students and the general population). The results paralleled those of Cronbach’s alpha, indicating that measurements obtained from general population samples demonstrated higher reliability than those from university student samples.

A relatively large number of moderator analyses were conducted to explore potential sources of heterogeneity. As noted in the meta-analytic literature, although multiple testing can increase the risk of Type I error, there is no consensus on how this issue should be handled in subgroup analyses or meta-regression (Borenstein et al., 2009). Accordingly, rather than applying a uniform multiplicity correction across all tests, moderator results were interpreted cautiously and in context, with emphasis placed on the consistency, direction, and theoretical plausibility of effects rather than on isolated *p*-values. This approach is consistent with recommendations for exploratory moderator analyses in meta-analysis.

The findings indicate that the BPS demonstrates consistently high internal consistency across both Cronbach’s alpha and McDonald’s omega coefficients, providing convergent evidence for the scale’s reliability. Nonetheless, while reliability was sufficient for the majority of research objectives, greater caution is recommended in high-stakes or high-risk contexts where measurement precision is crucial. It is important to note that reliability constitutes a necessary but not sufficient condition for accurate measurement. In order to meaningfully interpret the data and make practical decisions, there is also a need for evidence of validity. However, the present RGMA did not address this additional aspect. The moderator’s analysis underscores the necessity for careful interpretation, suggesting that greater emphasis should be placed on the consistency and theoretical plausibility of observed patterns rather than on isolated significance tests. The results obtained for alpha and omega are largely compatible, thereby reinforcing confidence in the internal consistency of the BPS. However, they concurrently highlight the broader measurement considerations that extend beyond reliability alone.

### Limitations

In the present study, pooled reliability estimates were calculated, and subsequent analyses were conducted based on all reliability coefficients, regardless of the specific research context. However, the intended purpose of the instrument within each study, such as providing evidence for construct validity versus supporting decision-making about individuals, was not taken into account. Considering that the BPS has been linked to various health outcomes, including insomnia, obesity, and diabetes, it is important to recognize that future RGMA analyses of BPS should consider the specific contexts and applications of the scale. Conducting subgroup analyses based on these contextual distinctions may provide more nuanced insights into the reliability of the instrument in relation to its uses in different fields, including psychological and clinical settings.

### Implications

The findings of this study have important implications for both research and applied contexts. Although pooled reliability estimates were examined across all studies regardless of their specific context, the results highlight that certain moderators, such as age, region, and sample type, can meaningfully influence reliability outcomes. This suggests that researchers and practitioners should consider these factors when interpreting BPS scores, particularly in high-stakes or clinical contexts where decisions may have significant consequences.

With regard to prospective research, the non-significant moderators in this meta-analysis, including publication type, language, sample size, mean and standard deviation of BPS scores, and percentage of women, imply that these factors may have a negligible influence on reliability. Nevertheless, future studies could further explore their potential effects. Investigating the BPS in novel cultural, clinical, or applied settings may help identify conditions under which these variables could become more relevant. This would ultimately improve the generalizability and interpretability of the scale across diverse populations.

## Conclusion

This meta-analysis aimed to comprehensively review the reliability of the Bedtime Procrastination Scale (BPS) scores across diverse cultural and linguistic samples, considering methodological characteristics. The study demonstrated that the BPS exhibits strong internal consistency overall, including across gender, different versions of the scale, publication types, and sample sizes. It is vital for future studies to continue looking at the BPS in different research settings, such as clinical and medical decision-making, as well as large-scale empirical research, to look for ways to improve the accuracy and practical use of the tool in a range of research and applied situations.

## Data Availability

The data that support the findings of this study are openly available in the Open Science Framework (OSF) at https://doi.org/10.17605/OSF.IO/4YD5H.

## Footnotes

1 Bonett’s alpha bias transformation yields negative values because it is based on the logarithmic transformation of (1 − *α*). For ease of presentation, the *metafor* package reports the absolute values of the transformed coefficients. Accordingly, the actual transformed estimates corresponding to the reported pooled effect and confidence limits are −1.9286, −1.8525, and −2.0046, respectively. All *τ* and *τ* estimates are likewise expressed on the transformed scale.
