# Supplementary material for: A meta-analytic reliability generalization study of the Bedtime Procrastination Scale
Source: Front Psychol. 2026 Feb 18;17:1709258. doi: 10.3389/fpsyg.2026.1709258 (PMC12958060; doi:10.3389/fpsyg.2026.1709258)
Supplement: Supplementary file 1 [file Supplementary_file_1.pdf]

Supplementary Figures and Tables

Figure S1. Trim-and-fill funnel plot of Cronbach's alpha (Bonett-transformed) for the BPS

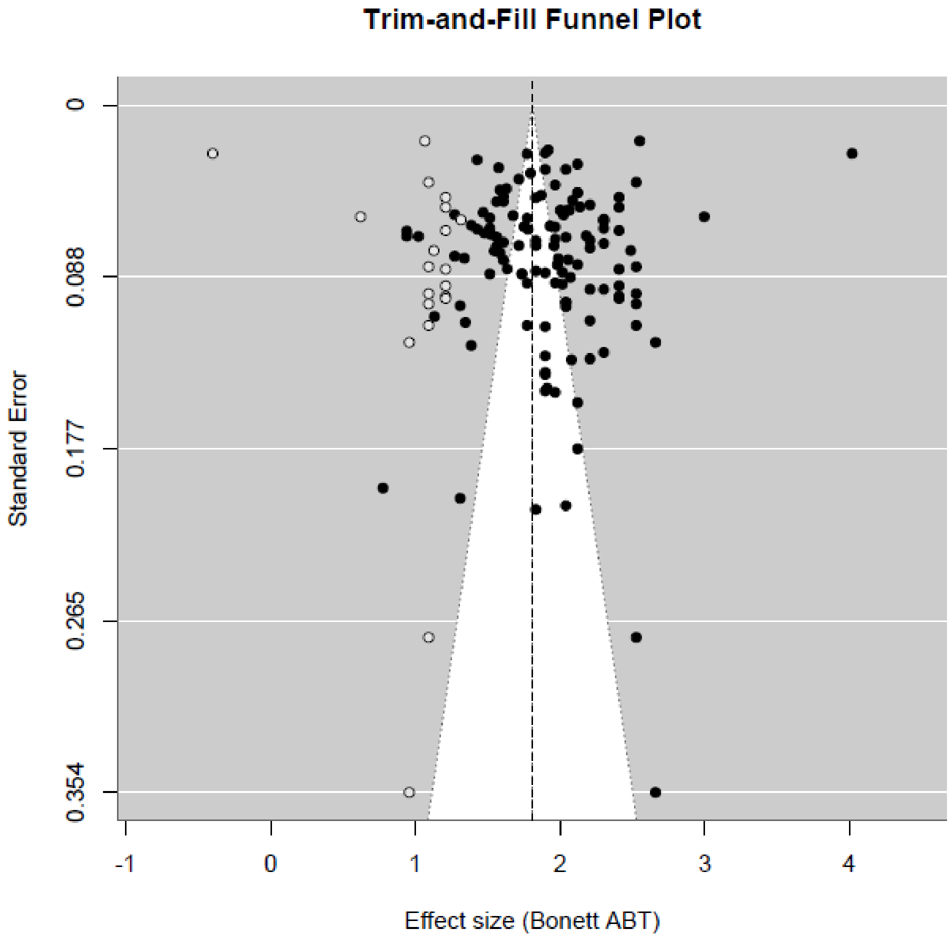

Figure S2. PET scatter plot of Cronbach's alpha (Bonett-transformed) for the BPS

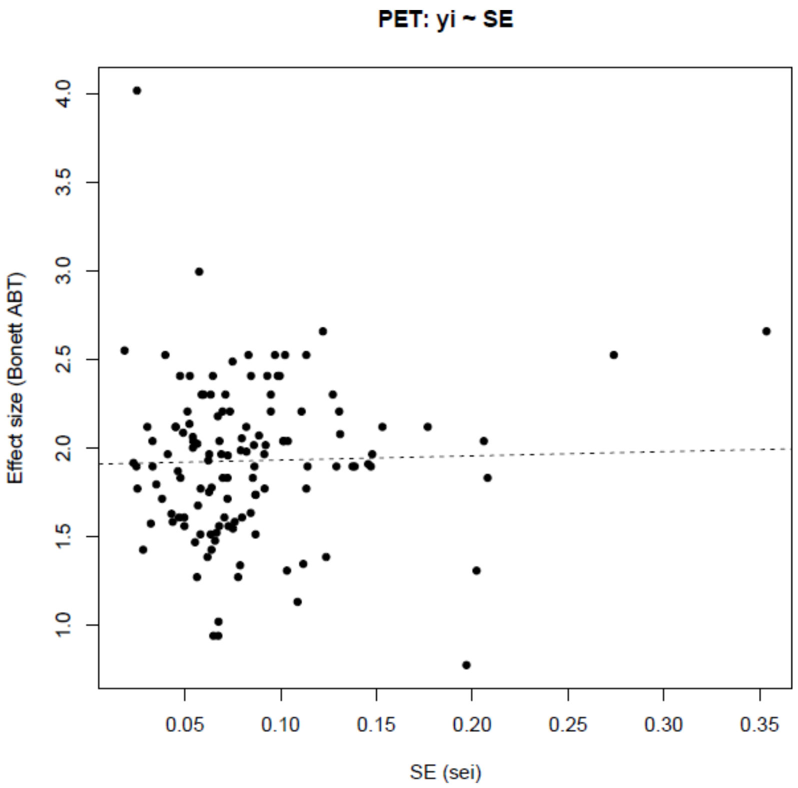

Figure S3. PEESE scatter plot of Cronbach's alpha (Bonett-transformed) for the BPS

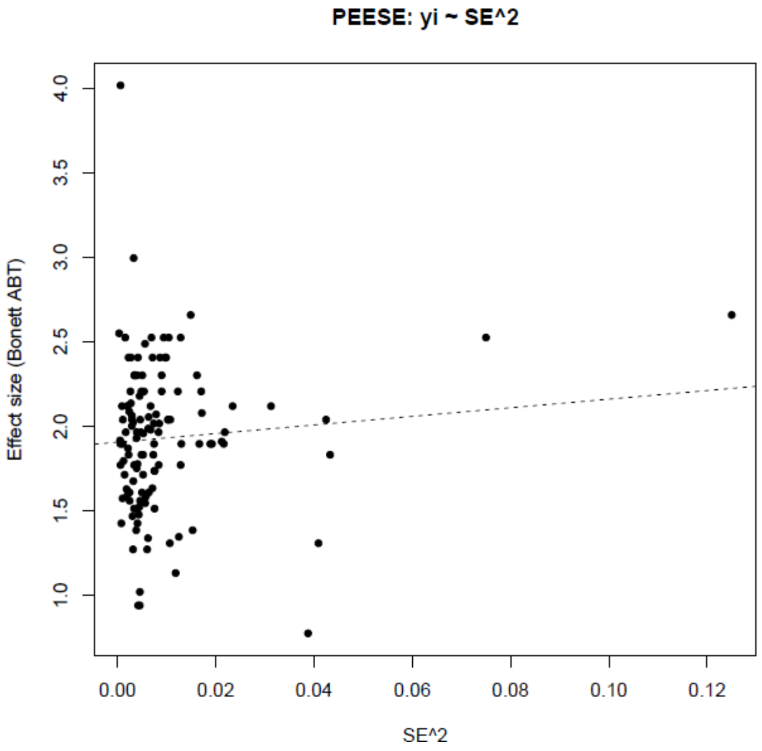

Figure S4 **Reliability Generalization of BPS (Cronbach's alpha) − Forest Plot**

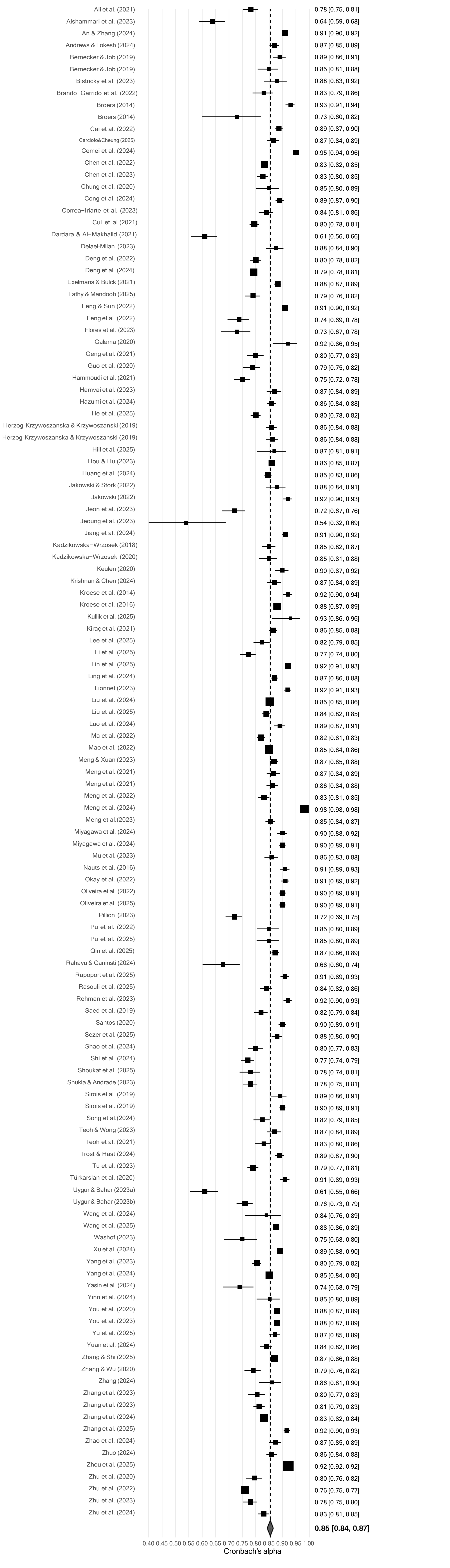

Figure S5. Normal Q-Q Plot of Standardized Residuals for Cronbach's alpha

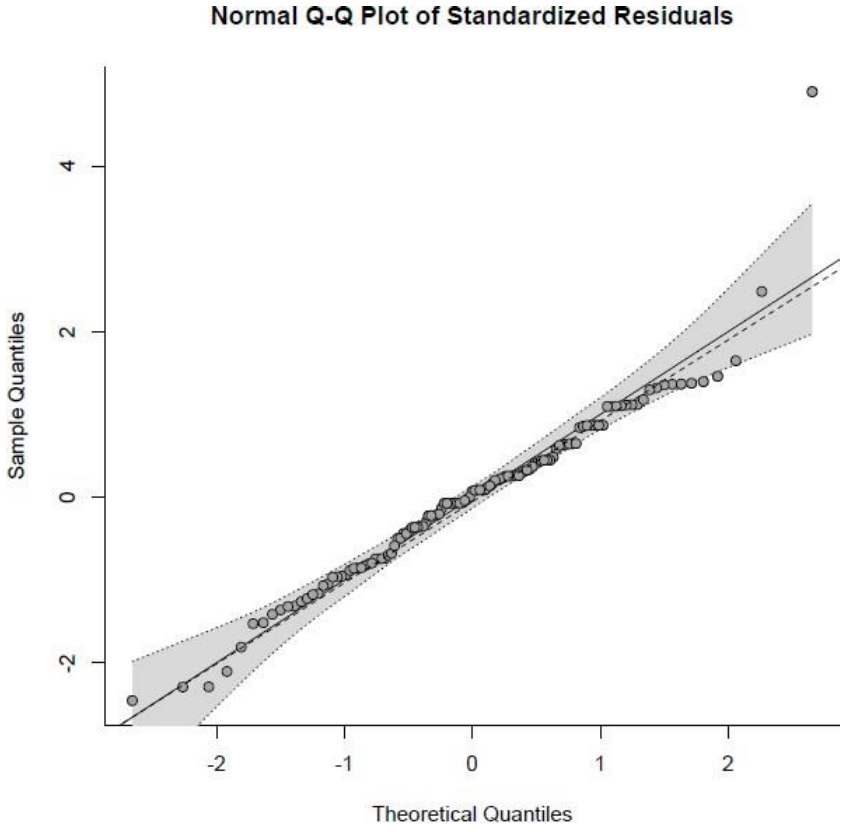

Figure S6. Meta-regression of Cronbach's alpha on mean age

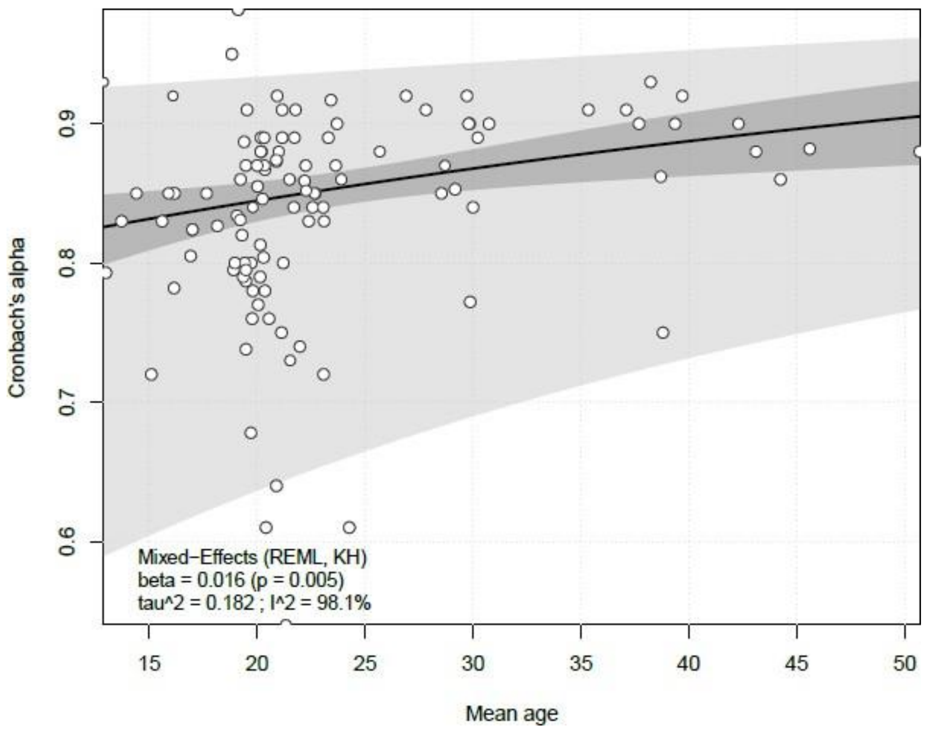

Figure S7. Meta-regression of Cronbach's alpha on age variability (sd of age)

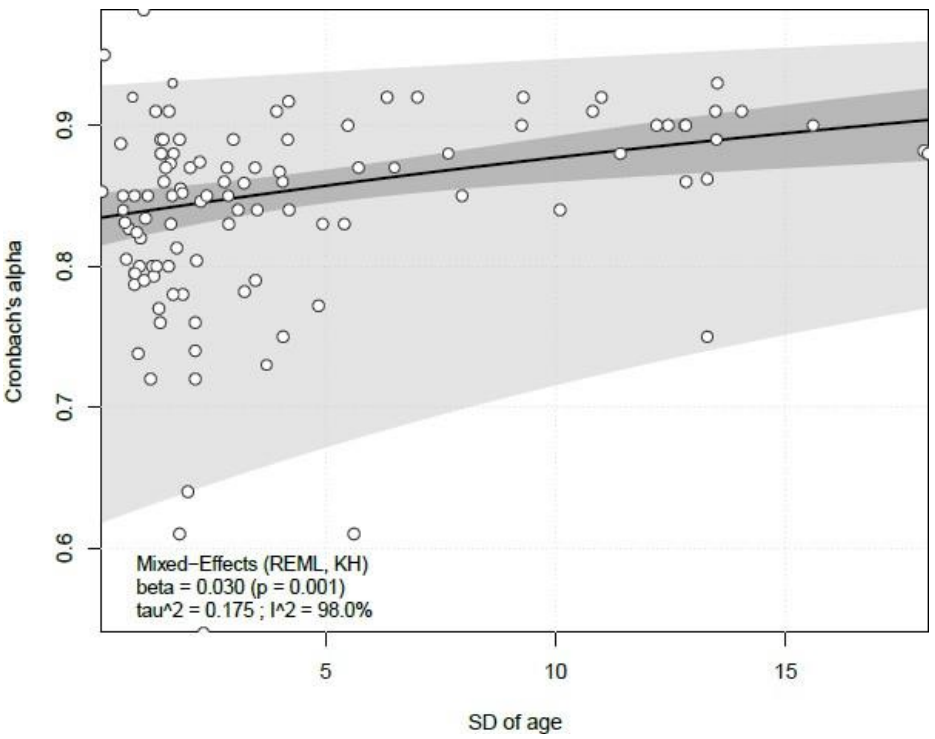

Table S1. Pairwise comparisons (Sample group; Bonferroni-adjusted)

group1	k1	alpha1	group2	k2	alpha2	diff_alpha	Z	*p_adj*
adolescent	12	0.825	general population	33	0.882	−0.057	−2.741	0.018
adolescent	12	0.825	university students	76	0.849	−0.025	−1.142	0.760
general population	33	0.882	university students	76	0.849	+0.032	2.789	0.016

Note. Tests on ABT scale; values shown as α for interpretability. *p*-values adjusted by Bonferroni**.**

Table S2. Proportion of variance explained (R^2) for Cronbach's alpha

Moderator	k_total	G	tau^2_total	tau^2_within	c	R^2 (%)
Region (Asia vs Europe)	109	2	0.175753	0.168586	0.041	4.1
Sample group	121	3	0.177919	0.164541	0.075	7.5

Note. $R^2 = 1 − (T^2$_within / T^2_total), quantifying the proportion of between-study (true) variance explained by the moderator(s). Estimates use the common-τ^2 framework (REML).

Figure S8. Trim-and-fill funnel plot of McDonald's omega (Bonett-transformed) for the BPS

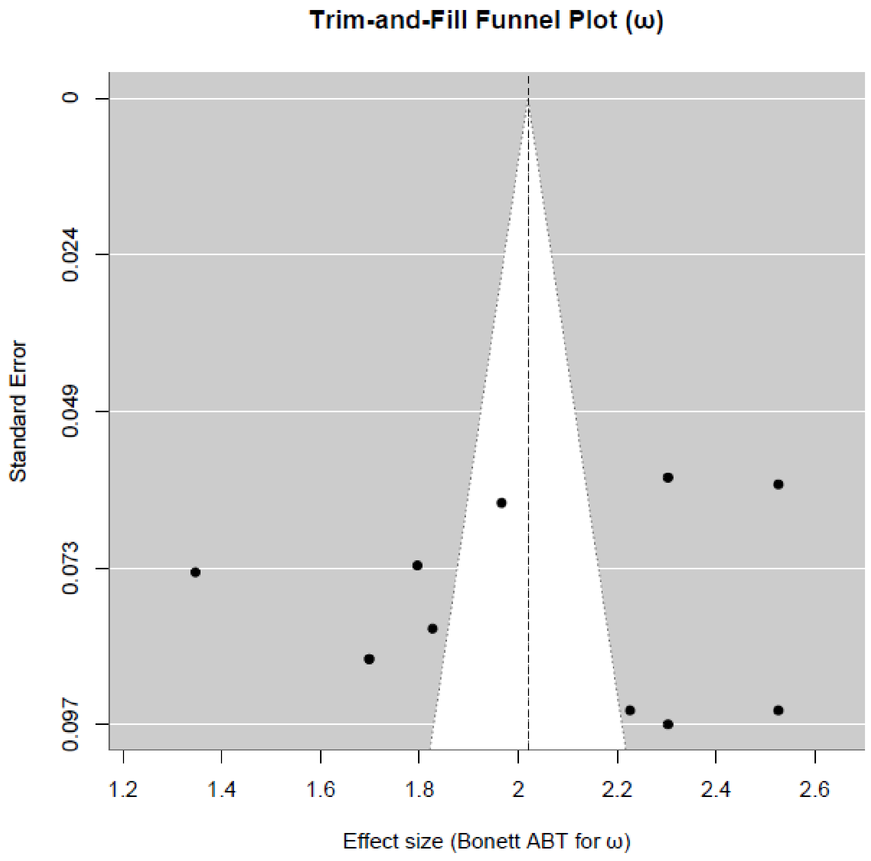

Figure S9. PET scatter plot of McDonald's omega (Bonett-transformed) for the BPS

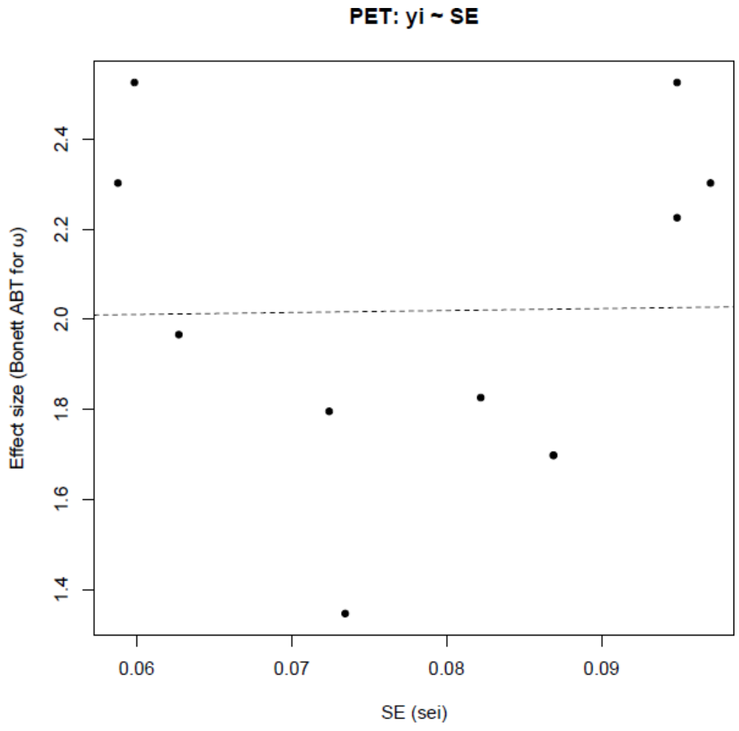

Figure S10. PEESE scatter plot of McDonald's omega (Bonett-transformed) for the BPS

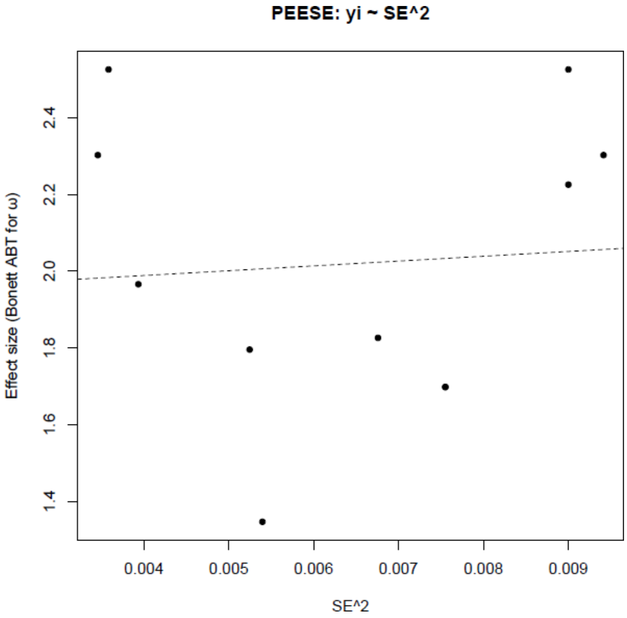

Figure S11. Normal Q-Q Plot of Standardized Residuals for McDonald's Omega

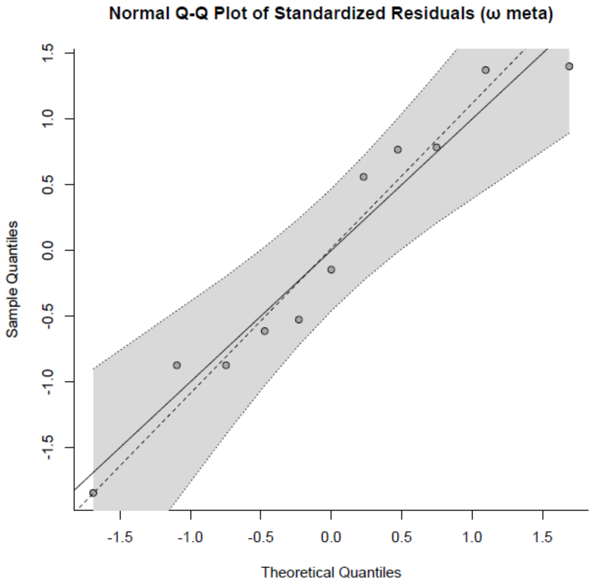

Table S3. Proportion of variance explained (R^2) for Mcdonald's omega

**Moderator**	**k_total**	G	**tau²_total**	**tau²_within**	**c**	**R^2 (%)**
Sample group (omega)	10	2	0.14423	0.09418	0.347011	34.7

Note. $R^2 = 1 - (T^2\_within / T^2\_total)$, quantifying the proportion of between-study (true) variance explained by the moderator. Estimates use the common-τ^2 framework (REML).
